# The Discovery of *N* ^2^,*N* ^2^‑Dimethylguanine Hydrolases Unravels General Molecular Principles of Enzyme Evolvability and Promiscuity

**DOI:** 10.1021/acscatal.6c00436

**Published:** 2026-03-23

**Authors:** Lukas Drexler, Cristina Duran, Sílvia Osuna, Reinhard Sterner

**Affiliations:** 1 Institute of Biophysics and Physical Biochemistry, Regensburg Center for Biochemistry, University of Regensburg, Regensburg D-93040, Germany; 2 Institut de Química Computacional i Catàlisi(IQCC) and Departament de Química,Universitat de Girona, Girona 17003, Spain; 3 ICREA, Barcelona 08010, Spain

**Keywords:** enzyme catalysis, enzyme evolution, promiscuity, *N* ^2^,*N* ^2^-dimethylguanine hydrolase, amidohydrolase superfamily, shortest path map analysis, conformational heterogeneity, xenobiotics

## Abstract

The widespread use of xenobiotics has driven the rapid emergence of microbial degradation pathways. A prominent example is enzymes involved in the catabolism of the herbicide atrazine, which have evolved within the last few decades. Recently, we provided evidence that the second enzyme of the atrazine biodegradation pathway, hydroxyatrazine ethylaminohydrolase (AtzB), has evolved from a progenitor enzyme of the amidohydrolase superfamily (AtzB-CQNN) with guanine deaminase (GuaD) activity. However, the catalytic efficiency for guanine hydrolysis by AtzB-CQNN is several orders of magnitude lower than that of prototypical GuaDs. In this study, we report a much higher catalytic efficiency of AtzB-CQNN for the hydrolysis of the guanine analogue *N* ^2^,*N* ^2^-dimethylguanine (*k*_cat_/*K*_M_ ∼10^5^–10^6^ M^–1^s^–1^). This enzymatic activity has not been described up to now and appears to be the native function of AtzB-CQNN, as well as that of several AtzB homologues termed NdmH. An active site alanine scan of an NdmH enzyme allowed us to identify residues important for substrate binding and catalysis and to propose an enzymatic reaction mechanism. The comparative characterization of NdmHs and canonical GuaDs revealed an extended substrate scope and high evolvability of NdmH enzymes. A comprehensive computational evaluation, including conservation, covariance, and flexibility studies, as well as conformational landscape reconstruction and correlation-based shortest path map analysis, showed that this enhanced substrate promiscuity and evolvability of NdmHs compared with GuaDs are linked to a higher structural heterogeneity of the active site, which facilitates their functional diversification to act on xenobiotics.

## Graphical abstract

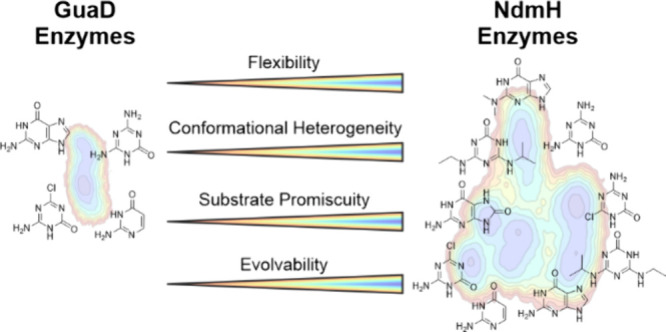

## Introduction

The extensive release of xenobiotics, which were not present in nature before human interference, has significantly affected the global ecosystem. Atrazine, an example of such an anthropogenic compound, has been used in agriculture as a herbicide since the 1950s, initially leading to its accumulation in the environment. Later, soil concentrations of atrazine and related *s*-triazines were found to have decreased drastically, indicating microbial degradation of these xenobiotics. Indeed, in the 1990s, *Pseudomonas sp.* strain ADP was identified as the first bacterium capable of completely breaking down atrazine. The responsible enzymes, atrazine chlorohydrolase (AtzA), hydroxyatrazine ethylaminohydrolase (AtzB), and *N*-isopropylammelide isopropylaminohydrolase (AtzC) (Figure 1A), are assumed to be recent evolutionary acquisitions that have emerged from promiscuous side activities of existing enzymes driven by the appearance of atrazine in the environment.^1–9^ AtzA, AtzB, and AtzC are members of the amidohydrolase superfamily (AHS) and possess the frequently encountered (βα)_8_-barrel fold whose core is composed of eight β-strands forming a curved central parallel barrel, which is enveloped by eight α-helices (Figure 1B). On one pole of the barrel, the connecting βα-loops contain residues important for substrate and metal binding and catalysis, whereas the opposite pole contributes to conformational stability.^10,11^ This structural separation of a catalytic pole from a stability pole allows for activity changes without compromising stability, which is highly beneficial for the evolution of novel enzyme functions. The AHS contains more than one million proteins, constituting one of the functionally most important and diverse enzyme superfamilies: AHS enzymes usually catalyze amide or ester hydrolysis at carbon and phosphorus centers in a metal-dependent manner, and many of them show promiscuous side activities.^12–19^ Due to this promiscuity, their frequent occurrence, their catalytic versatility, and their polar fold architecture, AHS members provide an excellent opportunity to study the evolution of novel enzymes from ancient progenitors.

**Figure 1:**
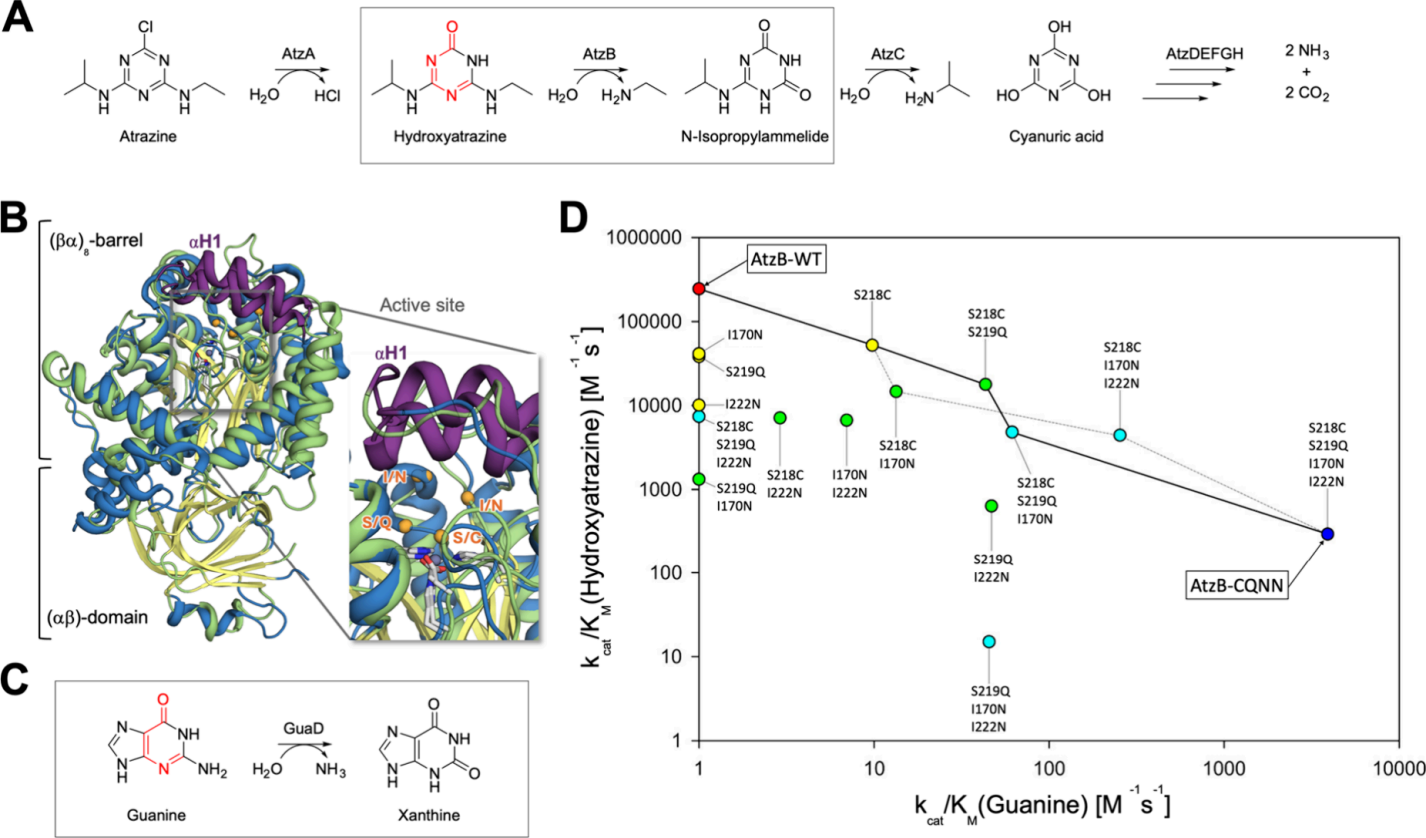
(A) The Atz pathway comprises the recently evolved enzymes AtzABC, degrading atrazine to cyanuric acid, and AtzDEFGH, degrading cyanuric acid to ammonia and carbon dioxide. Reproduced from ref 24. Available under a CC-BY 4.0 license. 2026 Wiley-VCH. (B) AHS enzymes are composed of a (βα)_8_-barrel, which harbors the active site on the catalytic pole of the barrel, while the opposite stability pole is connected to an (αβ)-domain. Provided is an overlay of *E. coli* guanine deaminase (*ec*GuaD, PDB: 6OHB) and an AlphaFold3 model of AtzB (RMSD value of 2.2 Å), together with a zoom of the active site pocket indicating the position of the SSII (CQNN) motif of AtzB (homologues). (C) GuaDs catalyze the hydrolytic cleavage of guanine to xanthine. Reproduced from ref 24. Available under a CC-BY 4.0 license. 2026 Wiley-VCH. (D) Substrate specificity changes in the entire sequence space connecting AtzB-CQNN (rightmost variant, dark blue) with wild-type AtzB (leftmost variant, red) as recently determined.^9^ Bold and dotted lines denote plausible evolutionary trajectories along which the guanine deaminase ancestor AtzB-CQNN could have evolved to gain increased catalytic efficiency (*k*_cat_/*K*_M_) for hydroxyatrazine, finally resulting in wild-type AtzB. Reproduced from ref 9. 2023 American Chemical Society.

Along these lines, we have previously retraced the rapid evolution of AtzB from a putative ancestor.^9^ Our investigations revealed a low promiscuous guanine deaminase (GuaD) activity for AtzB (Figure 1C), while the closest AtzB homologues showed a decent guanine deaminase activity and a low promiscuous hydroxyatrazine ethylaminohydrolase activity. Based on sequence comparisons with these AtzB homologues, the variant AtzB-CQNN with four active site exchanges was generated: SSII in AtzB was converted to CQNN as present in the AtzB homologues (Figure 1B, D). Remarkably, AtzB-CQNN showed a GuaD activity that was comparable to those of the AtzB homologues and 3 orders of magnitude higher compared with AtzB, thus representing an evolutionarily plausible predecessor.

However, the catalytic efficiencies for the conversion of guanine to xanthine (*k*_cat_/*K*_M_ ∼10^3^ M^–1^s^–1^) of the AtzB homologues and the putative progenitor enzyme AtzB-CQNN are still 2–3 orders of magnitude lower compared with well-characterized physiological guanine deaminases, such as the one from *Escherichia coli* (*ec*GuaD)^20^ or other species.^21–23^ Consequently, we hypothesized that guanine deamination might not be the primary function of the AtzB homologues and AtzB-CQNN, which prompted us to search for their native substrates by screening multiple compounds related to guanine. In doing so, we characterized a novel enzymatic activity, *N* ^2^,*N* ^2^-dimethylguanine hydrolysis, for the first time and assigned this function as native to AtzB-CQNN and to several AtzB homologues, which were subsequently termed NdmH (*N* ^2^,*N* ^2^-dimethylguanine hydrolase). Moreover, comparative experimental and computational analyses between GuaD and NdmH allowed us to uncover the molecular characteristics underlying increased enzymatic promiscuity of NdmHs and to identify mechanisms that shape their evolvability.

## Results and Discussion

### Analysis of Guanine Deaminase Clusters

In the search for the native function of the AtzB homologues and the main activity of the putative AtzB ancestor AtzB-CQNN, we first generated a sequence similarity network (SSN) for 11,000 unique sequences based on a recently analyzed AHS subtype III cluster of guanine deaminases.^24^ This analysis revealed the emergence of two guanine deaminase clusters at a 35.0% sequence identity cutoff (Figure S1). One cluster harbored AtzB, *pa*8-OxoGuaD (8-oxoguanine deaminase from *Pseudomonas aeruginosa*), and two previously characterized AtzB homologues (AtzB_Hom_Hal from *Haliea sp*. SAOS-164, henceforth termed '*hs*NdmH'; AtzB_Hom_Pleo from *Pleomorphomonas oryzae*, henceforth termed ‘*po*NdmH’),^9^ and the uncharacterized MBD1203459 from *Rhodobacteraceae bacterium* (henceforth termed ‘*rb*NdmH’) (Figure S1, AtzB cluster). The other cluster harbored *ec*GuaD and two sequences (WP_135441580 from *Haliea sp*. SAOS-164, henceforth termed '*hs*GuaD'; WP_026789444 from *Pleomorphomonas oryzae*, henceforth termed '*po*GuaD') (Figure S1, *ec*GuaD cluster). To compare the two clusters regarding their catalytic efficiencies for the hydrolysis of guanine, we performed steady-state kinetic measurements (Figure S2). While enzymes from the AtzB cluster showed low-to-moderate guanine deaminase activities (*k*_cat_/*K*_M_ ≤ 2.2 × 10^3^ M^–1^s^–1^), enzymes from the *ec*GuaD cluster hydrolyzed guanine with catalytic efficiencies that are higher by several orders of magnitude (*k*_cat_/*K*_M_ ∼10^5^–10^6^ M^–1^s^–1^) (Figure 2A). The strongly increased guanine deaminase activities of *po*GuaD and *hs*GuaD compared with those of *po*NdmH and *hs*NdmH, respectively, indicate that the members of the *ec*GuaD cluster most likely represent the physiological guanine deaminases in the respective species. Moreover, these findings further reinforce our hypothesis that the observed guanine deaminase activities of AtzB cluster enzymes might not represent their native function.

**Figure 2:**
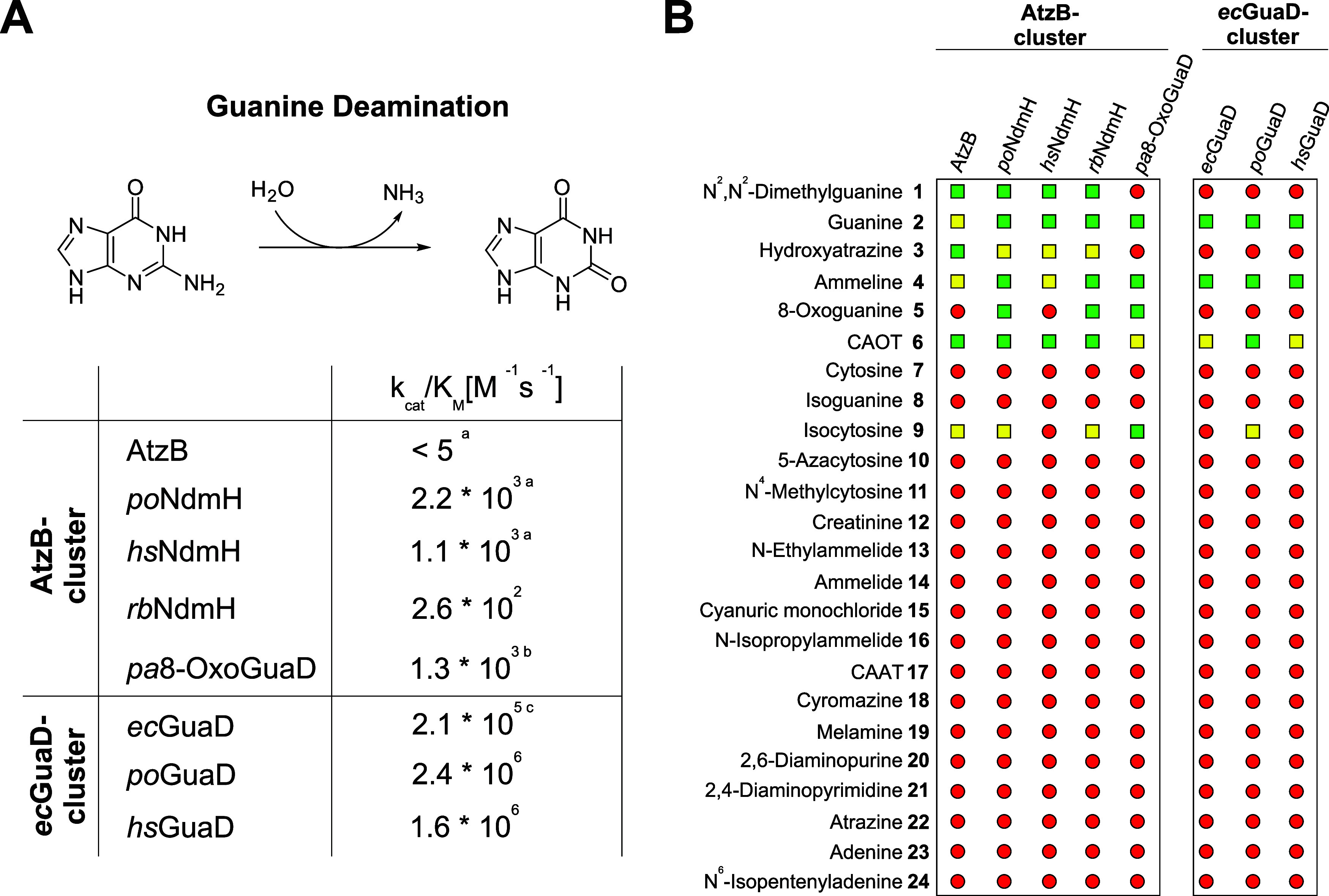
(A) Catalytic efficiencies (*k*_cat_/*K*_M_) for the deamination of guanine as determined by steady-state kinetics (cf. Figure S2). ^a^Values for AtzB, *po*NdmH, and *hs*NdmH were taken from our previous study.^9^ ^b,c^Values for *pa*8-OxoGuaD^14^ and *ec*GuaD^20^ were reported elsewhere. *ec*GuaD, *po*GuaD, and *hs*GuaD, which are part of the same cluster in the SSN (cf. Figure S1), show catalytic efficiencies that are 2–3 orders of magnitude higher compared with those of representatives from the second SSN cluster, including AtzB and its homologues. (B) Overview of the entire panel of substrates that can be converted by enzymes of the respective cluster. These substrate scopes were identified by HPLC-based enzyme assays,^24^ and the chemical formula for the catalyzed reactions is shown in Figure S3. Light green squares indicate enzyme–substrate pairs for which a total turnover of 500 μM substrate by 2 μM enzyme was detected after 24 h at 25 °C. Yellow squares indicate enzyme–substrate pairs for which partial substrate turnover was detected. Red circles indicate enzyme–substrate pairs for which no turnover was detected. Substrates 1–6 and 9 were recently^24^ shown to be hydrolyzed by the analyzed enzymes via a 1,6 nucleophilic conjugate addition. Reproduced from ref 24. Available under a CC-BY 4.0 license. 2026 Wiley-VCH.

To identify the native substrate of *hs*NdmH, *po*NdmH, and *rb*NdmH, we refined our previously published HPLC-based substrate screenings^24^ by distinguishing between total substrate turnover (Figure 2B, green boxes) and partial substrate turnover (Figure 2B, yellow boxes) by the respective enzymes.

This analysis revealed that members of the AtzB cluster possess an enlarged substrate scope, indicating enhanced promiscuity. Within this subgroup, catalysis of nine distinct reactions was observed (Figure 2B, left; Figure S3A), whereas within the *ec*GuaD cluster, catalysis of four distinct reactions was observed (Figure 2B, right; Figure S3B). Specifically, a given enzyme from the AtzB cluster, on average, can convert 6.0 distinct substrates, whereas a given enzyme from the *ec*GuaD cluster, on average, can convert 3.3 distinct substrates. The altered degree of promiscuity becomes even more evident when examining the cleaved leaving groups, which include −NH_2_, −NHR (R = alkyl), −NR^1^R^2^ (R^1^, R^2^ = alkyl), and −Cl in the case of AtzB cluster enzymes (Figure S3A), whereas −NH_2_ is the only one observed in the case of *ec*GuaD cluster enzymes (Figure S3B). We assume that the latter enzymes constitute physiological guanine deaminases involved in primary metabolism and regulating the nucleobase pool, while the former enzymes participate in the degradation of secondary metabolites, such as guanine analogues (Figure 2B, compounds **1**, **5**, and **9**) and xenobiotics (Figure 2B, compounds **3**, **4**, and **6**). Indeed, for *pa*8-OxoGuaD, which clusters within the AtzB-subgroup in the SSN, the native function was previously shown to be the deamination of 8-oxoguanine, thereby aiding in the removal of this mutagenic guanine analogue.^14^ Importantly, the guanine analogue *N* ^2^,*N* ^2^-dimethylguanine (Figure 2B, compound **1**) was the only naturally occurring compound (in addition to guanine) that was fully degraded by *po*NdmH, *hs*NdmH, and *rb*NdmH, which prompted us to hypothesize that this is the native substrate of these AtzB homologues.

### Characterization of *N* ^2^,*N* ^2^-Dimethylguanine Hydrolases (NdmHs)

To confirm that *N* ^2^,*N* ^2^-dimethylguanine is the main substrate of *po*NdmH, *hs*NdmH, and *rb*NdmH, we first verified xanthine as a product of enzymatic *N* ^2^,*N* ^2^-dimethylguanine hydrolysis by HPLC (Figure 3A) and UV spectroscopy (Figure 3B). Moreover, time-dependent UV-spectroscopic analysis of the enzymatic reaction revealed the presence of isosbestic points at 266 and 279 nm (Figure 3C), indicative of a one-step reaction progress as shown in Figure 3D. We determined the differential molar extinction coefficient at 294 nm (Figure S4; *N* ^2^,*N* ^2^-dimethylguanine-xanthine: Δε_294_ = 3187 M^–1^cm^–1^) for subsequent steady-state kinetic experiments (Figure S5). These measurements revealed high catalytic efficiencies for the hydrolysis of *N* ^2^,*N* ^2^-dimethylguanine (*k*_cat_/*K*_M_ ∼10^5^–10^6^ M^–1^s^–1^), for both the AtzB homologues (*po*NdmH, *hs*NdmH, and *rb*NdmH) and the putative AtzB ancestor (AtzB-CQNN) (Figure 3D). These values, which are each 150–3600-fold higher compared with those for guanine (Table S1), indicate that the AtzB homologues are the first ever discovered native *N* ^2^,*N* ^2^-dimethylguanine hydrolases (NdmHs) and that AtzB has evolved from a predecessor with this activity as outlined in a subchapter below. The *k*_cat_/*K*_M_ values determined are of the same order of magnitude as those for similar AHS enzymes, which prefer alkylated nucleobases as their main substrates over the nonalkylated ones (e.g.: 5-methylcytosine deaminase,^25^ cytokinin deaminase,^26^ and *N*-6-methyladenine deaminase^27^).

**Figure 3:**
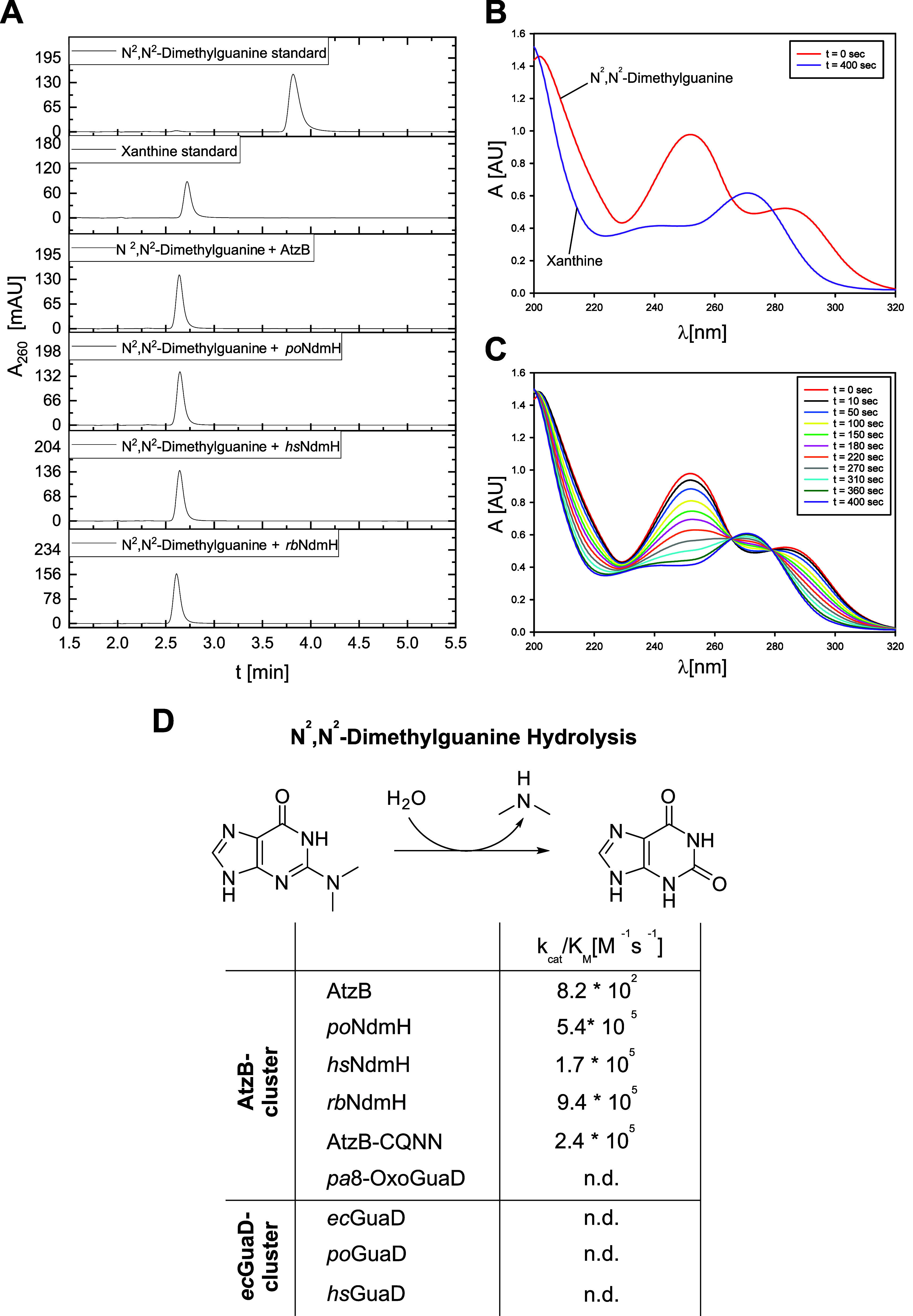
(A) Incubation of *N* ^2^,*N* ^2^-dimethylguanine (500 μM) with AtzB, *po*NdmH, *hs*NdmH, or *rb*NdmH (2 μM) in 50 mM potassium phosphate buffer (pH 7.5) for 24 h at 25 °C, resulting in the formation of xanthine as indicated by HPLC-based analysis of the reaction products at 260 nm. (B) Absorbance spectra of the reaction mixtures of *N* ^2^,*N* ^2^-dimethylguanine (1 mM) in 50 mM potassium phosphate buffer (pH 7.5) before (*t* = 0 s) and after (*t* = 400 s) the addition of *po*NdmH (200 nM), resulting in the formation of xanthine. (C) Time-dependent UV-spectroscopic analysis of the same reaction mixtures, revealing the presence of isosbestic points (266 nm, 279 nm) that indicate a one-step reaction progress. (D) Catalytic efficiencies (*k*_cat_/*K*_M_) for the hydrolysis of *N* ^2^,*N* ^2^-dimethylguanine as determined by steady-state kinetics (cf. Figure S5); n.d.: no activity detectable.

Next, we determined the oligomerization states of the members of the AtzB cluster by analytical size-exclusion chromatography. These experiments showed that NdmH enzymes as well as AtzB and its putative progenitor AtzB-CQNN mainly occur as homodimers (Figure S6), similar to previously characterized physiological guanine deaminases.^28,29^ For further functional studies, *po*NdmH was chosen as an NdmH model system, as it is the enzyme with the highest catalytic activity that stems from a phylogenetically classified organism and thus will facilitate planned follow-up studies in the genomic context of NdmHs. First, we comprehensively analyzed the active site of *po*NdmH to identify catalytically essential residues and examine the underlying enzymatic reaction mechanism: Mutagenesis of the residues CQNN, as present in the AtzB homologues and AtzB-CQNN, to SSII, as present in AtzB, is accompanied by strongly impaired hydrolysis of *N* ^2^,*N* ^2^-dimethylguanine (cf. Figure 3D) and guanine (cf. Figure 1D).^9^ Remarkably, albeit not being in direct contact with the substrate, these CQNN residues (*po*NdmH: C213, Q214, N217, and N165; Figure 4A) were shown to influence the conformation of four substrate-flanking residues (*po*NdmH: W85, W93, Y98, and Y133; Figure 4A).^9^ We therefore generated *po*NdmH alanine single mutants of these four and three additional substrate-flanking residues, purified the corresponding proteins to homogeneity (Figure S7), and confirmed their structural integrity by far-UV CD spectroscopy (Figure S8). Then, we determined the catalytic parameters for the hydrolysis of *N* ^2^,*N* ^2^-dimethylguanine by steady-state enzyme kinetics (Figure S9) to assess the influence of each individual alanine mutation: Y98A, P300A, W93A, W85A, and Y133A showed 8-fold, 17-fold, 75-fold, 125-fold, and 2100-fold decreases in catalytic efficiency (Figure 4B), respectively, indicating a significant contribution of the wild-type residues to substrate binding and/or positioning for catalysis. No activity was detectable for Q74A as well as E243A, indicating the direct involvement of the wild-type residues in catalysis. Based on these findings and on our previous mechanistic analyses of guanine deaminases showing that catalysis proceeds via a 1,6 nucleophilic conjugate addition to a substrate,^24^ we conclude that hydrolysis of *N* ^2^,*N* ^2^-dimethylguanine by NdmH enzymes involves a catalytic machinery (Figure 4C, *po*NdmH: Q74, E243, H275, and D326) equivalent to that found in well-characterized guanine deaminases. To assess the sequence conservation of this catalytic machinery, we generated a multiple sequence alignment (MSA) for both the AtzB and *ec*GuaD clusters individually: Q74, E243, H275, and D326 show high sequence conservation of 86.3%/98.4%, 98.0%/99.9%, 99.0%/99.9%, and 99.2%/99.6% in the AtzB/*ec*GuaD cluster, respectively. Moreover, we analyzed the metal-binding motif among the studied NdmH enzymes (Figure S10), indicating the involvement of a divalent metal ion in catalysis as characteristic for AHS enzymes.^12,29,30^ Hence, we propose the following reaction mechanism for the mode of action of NdmH enzymes: The conserved D326, characteristic for deaminase enzymes of the AHS, deprotonates a metal-bound water nucleophile, and the proton is shuttled from D326 to E243 by the bridging H275 (Figure 4C-I). *N* ^2^,*N* ^2^-Dimethylguanine is protonated at the lone pair localized in the sp^2^ orbital of N3 by the adjacent E243, while the nucleophile attacks the C=N double bond of the substrate at the Bürgi–Dunitz angle (107°) through a 1,6 conjugate addition (Figure 4C-II).^24^ A new stereocenter is formed within the tetrahedral intermediate in which the exocyclic oxyanion is stabilized by Q74. E243 deprotonates N3 of the substrate and protonates the leaving group, resulting in a backflow of π-electrons (Figure 4C-III). Dimethylamine is released, and the product xanthine is formed (Figure 4C-IV).

**Figure 4:**
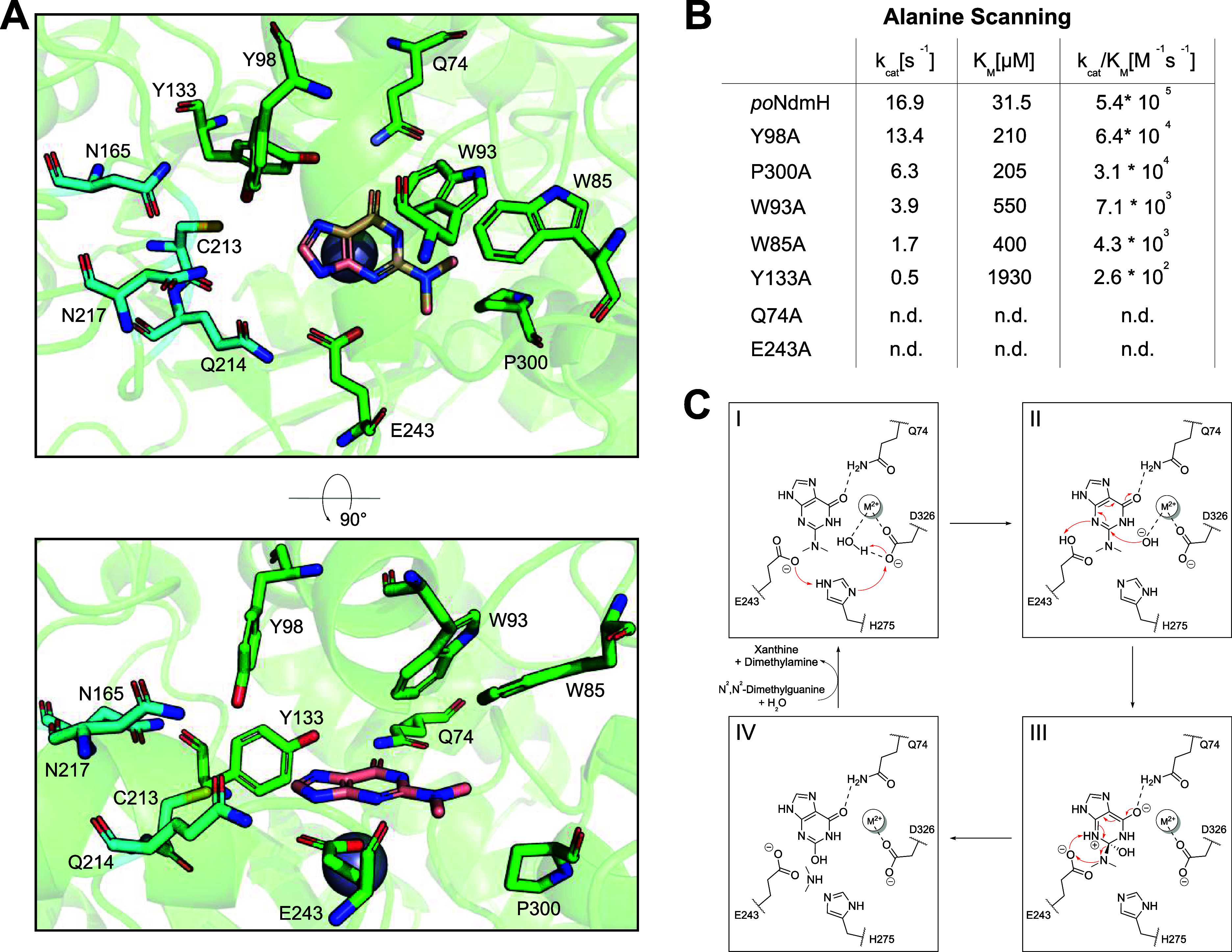
(A) AlphaFold structure of *po*NdmH representing the active site architecture of NdmH enzymes. The position of the divalent metal ion (gray sphere) and the substrate *N* ^2^,*N* ^2^-dimethylguanine (salmon sticks) was inferred from an overlay with *Saccharomyces cerevisiae* GuaD (PDB: 6OHA). The residues depicted as blue sticks correspond to the CQNN residues that are mutated to SSII in the course of the evolution of AtzB (Figure 1D). Further active site residues surrounding the substrate are shown as green sticks and were chosen for an alanine scan. (B) Alanine scanning of *po*NdmH active site residues and steady-state kinetic experiments (cf. Figure S9) revealed impaired or no detectable (n.d.) catalysis of *N* ^2^,*N* ^2^-dimethylguanine hydrolysis for all mutants. (C) Proposed reaction mechanism for the hydrolysis of *N* ^2^,*N* ^2^-dimethylguanine by NdmH enzymes can be divided into four steps: I: The water nucleophile is deprotonated by an aspartate (*po*NdmH: D326), and the proton is shuttled from this aspartate (D326) to a glutamate (*po*NdmH: E243) by a bridging histidine (*po*NdmH: H275). II: *N* ^2^,*N* ^2^-dimethylguanine is protonated by the glutamate (E243), and the nucleophile attacks the substrate’s C=N double bond at the Bürgi–Dunitz angle (107°) through a 1,6 nucleophilic conjugate addition. III: A new stereocenter is formed within the tetrahedral intermediate in which the oxyanion is stabilized by a glutamine (*po*NdmH: Q74). The glutamate (E243) deprotonates N3 and protonates the dimethylamine substituent, resulting in a backflow of π-electrons. IV: The leaving group is released, and the product xanthine is formed. Electron flows and divalent metal ions (most likely zinc; see Figure S10) are represented by red arrows and M^2+^, respectively. Reproduced from ref 24. Available under a CC-BY 4.0 license. 2026 Wiley-VCH.

### Investigating Enzyme Evolvability by Functional Conversion of NdmH Enzymes

Within previous research, we have retraced the evolution of AtzB by protein engineering.^9^ Although our initial results indicated that AtzB has evolved from a guanine deaminase ancestor, we showed above that the primary function of the proposed AtzB progenitor (AtzB-CQNN) and the AtzB homologues (*po*NdmH, *hs*NdmH, and *rb*NdmH) is the hydrolysis of *N* ^2^,*N* ^2^-dimethylguanine. This raises the question of whether AtzB-CQNN can still be considered a valid ancestor of AtzB, and by what evolutionary path it developed. To address this issue, we determined the catalytic parameters for *N* ^2^,*N* ^2^-dimethylguanine hydrolysis for all 14 previously analyzed evolutionary intermediary variants (cf. Figure 1D) connecting AtzB-CQNN and wild-type AtzB (Figure S11). This analysis showed that AtzB-CQNN is the variant possessing the highest *N* ^2^,*N* ^2^-dimethylguanine hydrolase activity and that it is connected by two evolutionary plausible trajectories (Figure 5A, bold and dotted lines) with wild-type AtzB, implying that it is indeed its evolutionary progenitor. Notably, the originally identified trajectories connecting guanine and hydroxyatrazine hydrolysis (Figure 5B, left trajectories) are virtually identical to these newly identified trajectories connecting *N* ^2^,*N* ^2^-dimethylguanine and hydroxyatrazine hydrolysis (Figure 5B, right trajectories) in terms of shape and order of the mutations. This is most likely due to the structural resemblance of guanine and *N* ^2^,*N* ^2^-dimethylguanine, which differ only in their leaving groups, and due to the remote location of the mutated residues relative to these leaving groups. Strikingly, however, *N* ^2^,*N* ^2^-dimethylguanine hydrolysis, compared with guanine hydrolysis, is elevated by several orders of magnitude for each analyzed AtzB variant (Figure 5B).

**Figure 5:**
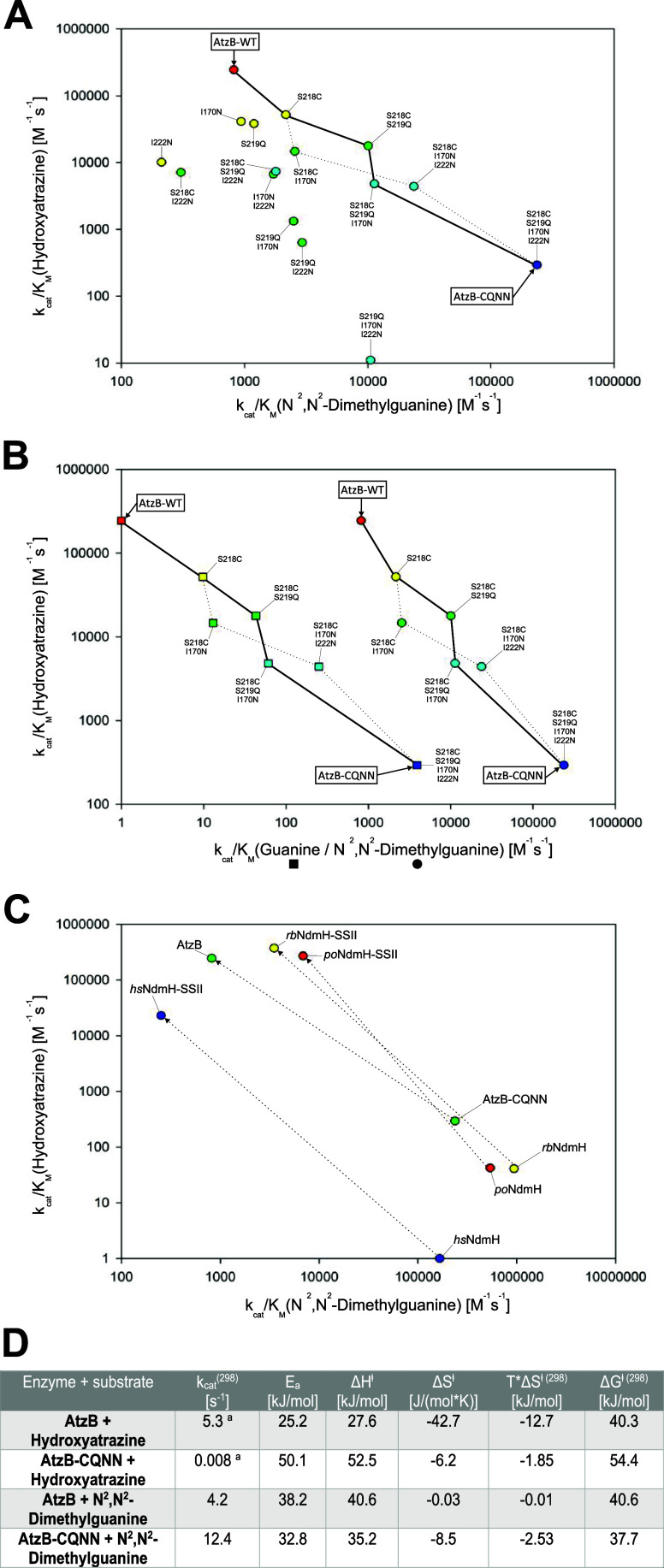
(A) Substrate specificity changes in the entire sequence space connecting wild-type AtzB (AtzB-WT) and its putative ancestor AtzB_S218C_S219Q_I170N_I222N (AtzB-CQNN) as indicated by plotting the catalytic efficiencies (*k*_cat_/*K*_M_) for hydroxyatrazine against *k*_cat_/*K*_M_ for *N* ^2^,*N* ^2^-dimethylguanine (cf. Figures S5 and S12). Colors denote the number of mutations relative to wild-type AtzB (0 = red; 1 = yellow; 2 = green; 3 = light blue; 4 = dark blue). Two evolutionarily plausible trajectories that lead from AtzB-CQNN to AtzB and feature similar activity trade-offs are shown as bold and dotted lines. (B) Catalytic efficiencies (*k*_cat_/*K*_M_) for hydroxyatrazine are plotted against *k*_cat_/*K*_M_ for guanine (boxes) and *N* ^2^,*N* ^2^-dimethylguanine (circles), respectively. The left trajectories corresponding to guanine hydrolysis (cf. Figure 1D)^9^ are identical to the right trajectories corresponding to *N* ^2^,*N* ^2^-dimethylguanine hydrolysis (cf. Figure 5A) in terms of shape and order of the mutations. However, values for *N* ^2^,*N* ^2^-dimethylguanine are elevated by several orders of magnitude for each analyzed enzyme variant. (C) Catalytic efficiencies (*k*_cat_/*K*_M_) for hydroxyatrazine are plotted against *k*_cat_/*K*_M_ for *N* ^2^,*N* ^2^-dimethylguanine (cf. Figure S13). For all NdmH-like enzymes (i.e., AtzB-CQNN, *po*NdmH, *hs*NdmH, and *rb*NdmH), exchange of the same set of residues (CQNN to SSII) yields highly efficient hydroxyatrazine hydrolases (i.e., AtzB, *po*NdmH-SSII, *hs*NdmH-SSII, and *rb*NdmH-SSII) demonstrating rapid specialist-to-specialist transitions. While not resulting in the best evolutionary end point (regarding hydroxyatrazine hydrolase activity), the AtzB-CQNN scaffold provided the highest initial side activity for hydroxyatrazine, thereby representing the best evolutionary starting point. (D) ^a^ *k*_cat_^(298)^ values for hydroxyatrazine were determined in our previous study,^9^ while *k*_cat_^(298)^ values for *N* ^2^,*N* ^2^-dimethylguanine were determined in the present study (c.f. Figure S5). Temperature-dependent activity measurements (278–303 K) for the hydrolysis of hydroxyatrazine and *N* ^2^,*N* ^2^-dimethylguanine by wild-type AtzB and AtzB-CQNN were performed in triplicates (cf. Figure S14) to determine the parameters of catalysis via the Arrhenius and Eyring equations.

Our findings clearly indicate that the hydroxyatrazine ethylaminohydrolase AtzB has evolved from its progenitor AtzB-CQNN, which is a highly active *N* ^2^,*N* ^2^-dimethylguanine hydrolase (NdmH). To assess the evolvability of NdmH enzymes in general, we aimed to imitate natural evolution by mutating the residues CQNN to SSII. To this end, we generated the mutants *po*NdmH-SSII (C213S, Q214S, N217I, and N165I), *hs*NdmH-SSII (C209S, Q210S, N213I, and N161I), and *rb*NdmH-SSII (C207S, Q208S, N211I, and N159I), purified the proteins to homogeneity (Figure S12), and determined the steady-state kinetic parameters for hydroxyatrazine and *N* ^2^,*N* ^2^-dimethylguanine hydrolysis (Figure S13). These measurements revealed a high evolvability of all analyzed NdmH enzymes and rapid specialist-to-specialist transitions (Figure 5C): Upon introduction of only four mutations, we were able to generate highly active hydroxyatrazine ethylaminohydrolases (*po*NdmH-SSII, *hs*NdmH-SSII, and *rb*NdmH-SSII) with catalytic efficiencies of 270,000, 23,000, and 370,000 M^–1^s^–1^, respectively, with accompanying negative trade-offs for *N* ^2^,*N* ^2^-dimethylguanine hydrolysis. Interestingly, two of these artificial enzymes (*po*NdmH-SSII, *rb*NdmH-SSII) outperformed the hydroxyatrazine ethylaminohydrolase activity of wild-type AtzB (240,000 M^–1^s^–1^), thereby indicating that alternative solutions for hydroxyatrazine hydrolysis could have readily evolved in nature. However, among all analyzed NdmH enzymes, AtzB-CQNN provided the highest initial promiscuous activity for hydroxyatrazine, hence constituting the best evolutionary starting point.

To gain further insights into this rapid functional conversion and to determine how evolutionary processes alter the rate of a chemical reaction, we investigated the turnover rates (*k*_cat_) for the hydrolysis of both hydroxyatrazine and *N* ^2^,*N* ^2^-dimethylguanine by AtzB and AtzB-CQNN in more detail. Steady-state kinetic measurements at 298 K indicate a pronounced increase of *k*_cat_ for hydroxyatrazine by a factor of 663 during the evolutionary optimization of AtzB starting from AtzB-CQNN,^9^ accompanied by a 3-fold reduction of *k*_cat_ for the original substrate *N* ^2^,*N* ^2^-dimethylguanine (Figure 5D). To analyze the origin of these changes in rate constants, we determined the activation energy (*E*_a_), the enthalpy of activation (Δ*H* ^⧧^), the entropy of activation (Δ*S* ^⧧^), and the Gibbs energy of activation (Δ*G* ^⧧^) by temperature-dependent activity measurements (278–303 K) and subsequent fitting of the data to both the Arrhenius and the Eyring equations (Figure S14). The values for these experimentally determined catalytic parameters are listed in Figure 5D. For other AHS members (e.g.: urease,^31^ adenosine deaminase,^32^ dihydroorotase,^33^ and uronate isomerase^34^), further (βα)_8_-barrel enzymes (e.g.: OMP decarboxylase,^35^ α-l-arabinofuranosidase^36^), and structurally unrelated enzymes catalyzing similar reactions (e.g.: cytidine deaminase^37^) comparable values within the same range were experimentally^31–33,35,37^ or computationally^34,36^ determined (*E*_a_ ∼21–54 kJ/mol; Δ*H* ^⧧^ ∼41–62 kJ/mol; T*Δ*S* ^⧧ (298)^ ∼−21–4 kJ/mol; Δ*G* ^⧧ (298)^ ∼47–72 kJ/mol). Furthermore, the average Δ*H* ^⧧^ of 39 kJ/mol observed in our measurements is in line with the average experimental Δ*H* ^⧧^ of ∼42 kJ/mol for enzyme-catalyzed reactions.^31^

For each analyzed enzyme–substrate pair, the enthalpic contribution Δ*H* ^⧧^ to Δ*G* ^⧧(298)^ exceeds the entropic contribution T*Δ*S* ^⧧(298)^ (Figure 5D), with an average fractional Δ*H* ^⧧^ contribution to Δ*G* ^⧧(298)^ of 89%. These results are in accordance with previous observations showing that the majority of enzymatic reactions are dominated by enthalpic contributions, with an average fractional Δ*H* ^⧧^ contribution of 77%.^31,38,39^ Next, we calculated the respective difference for each parameter for the evolutionary process AtzB-CQNN → AtzB (Figure S14). Notably, this revealed that the exceptionally high *k*_cat_ enhancement (663-fold for hydroxyatrazine) during the evolutionary optimization of AtzB from AtzB-CQNN is likewise dominated by Δ*H* ^⧧^ contributions.

In accordance with findings for other enzymes,^31,34,36,39,40^ we assume that the high enthalpic contributions observed in our experiments might be due to the electrostatic nature of catalysis, as showcased in Figure 4C for *N* ^2^,*N* ^2^-dimethylguanine and in Figure S15 for hydroxyatrazine: hydrolysis of both substrates involves a 1,6 nucleophilic conjugate addition^24^ that results in a tetrahedral intermediate (Figure 4C-III and Figure S15III) with greatly enhanced dipole moment (and minor dipole reorientation) compared with the respective substrate itself (Figure S16). The tetrahedral intermediate dipole is stabilized by a glutamate and a glutamine on its positive and negative poles (Figure 4C-III and Figure S15III), respectively. This specific active site environment stabilizes the intermediate’s dipole (resembling the transition state^41^) more than the substrate’s dipole and hence enables electric field catalysis (Figure S17).^38,39,42,43^ As the dipole orientations of *N* ^2^,*N* ^2^-dimethylguanine and hydroxyatrazine (Figure S16) differ only slightly and point in similar directions, the *k*_cat_-trade-offs between AtzB and AtzB-CQNN (Figure 5D) might be caused by a slight reorientation of the respective substrate within the active site’s electric field.^39,40^

### Comparative Analysis of Primary and Secondary Metabolic Enzymes to Unveil Determinants of Prominent Promiscuity and Evolvability

Sequence analyses showed that the newly identified *N* ^2^,*N* ^2^-dimethylguanine hydrolases do not cluster with canonical GuaDs in an SSN (cf. Figure S1). Members of both clusters were recently shown to catalyze the hydrolysis of substrates via a 1,6 nucleophilic conjugate addition and that this property defines the substrate scope of these enzymes since productive catalysis requires a 6-π-electron system within a substrate (e.g., Figure 1A/C, indicated in red).^24^ However, not all compounds fulfilling this criterion were used as substrates by all of these enzymes (Figure 2B), indicating that substrate promiscuity is influenced by the individual protein environment. Specifically, compared with canonical guanine deaminases of the *ec*GuaD cluster, NdmH enzymes exhibit an enlarged substrate scope (Figure 2B) capable of hydrolyzing chlorine- and alkylated leaving groups (Figure S3A). Moreover, we showed that AtzB has not evolved from a canonical GuaD but from an NdmH enzyme, all of which are highly evolvable (Figure 5C). We assume that enzymes from the canonical guanine deaminase cluster (*ec*GuaD cluster) show limited promiscuity and evolvability due to their crucial role in primary metabolism by regulating the nucleobase pool. In contrast, members of the AtzB cluster including AtzB, NdmH, and 8-OxoGuaD enzymes acting on secondary metabolites and xenobiotics might possess more flexible protein scaffolds tolerating function-changing mutations.

To further investigate this hypothesis and unveil the determinants of pronounced promiscuity and evolvability, we comparatively analyzed the two SSN clusters *in silico*. To this end, we analyzed the sequence conservation for the individual clusters using the constructed MSAs and mapped the respective conservation onto the 3D structure of representatives of each cluster via ConSurf^44^ (Figure 6, top panel). Moreover, VisualCMAT^45^ analysis of these MSAs allowed us to investigate sequence covariance within both clusters, i.e., we could identify coevolving positions in each protein scaffold (Figure 6, middle panel). A comparison of both clusters revealed a significantly higher degree of sequence conservation within the *ec*GuaD cluster. Specifically, the *ec*GuaD cluster showed a more pronounced conservation in the vicinity of the active site, while both clusters exhibited comparably well-conserved regions, particularly within the hydrophobic core of their β-barrels (Figure 6, top panel). Furthermore, covariance in the *ec*GuaD cluster was primarily observed in nonconserved regions of the protein scaffold, located remotely from the active site with sum of z-scores of up to 17.4 (Figure 6, middle panel). While these enzymes seem to tolerate sequence diversity, this variation is carefully segregated from the catalytic core: the preservation of the active site underscores the crucial role of these enzymes in the primary metabolism and regulation of the cellular nucleobase pool. Importantly, the evolutionary advantage of the *ec*GuaD cluster lies in the maintenance-of-function, ensuring a high degree of specificity and catalytic efficiency for their primary metabolic role. In contrast, for the AtzB cluster the evolutionary advantage is driven by the higher degree of promiscuity and ease of evolvability, allowing these enzymes to adapt and thus to act on a broader range of substrates, such as secondary metabolites as previously demonstrated for *pa*8-OxoGuaD.^14^ This is reflected in a lower sequence conservation of the active site environment and a strong covariance within a loop-helix-region with sum of *z*-scores of up to 23.1 (Figure 6, middle panel, framed with a black box), which shields the active site and is located in close proximity to the substrate’s leaving group.

**Figure 6:**
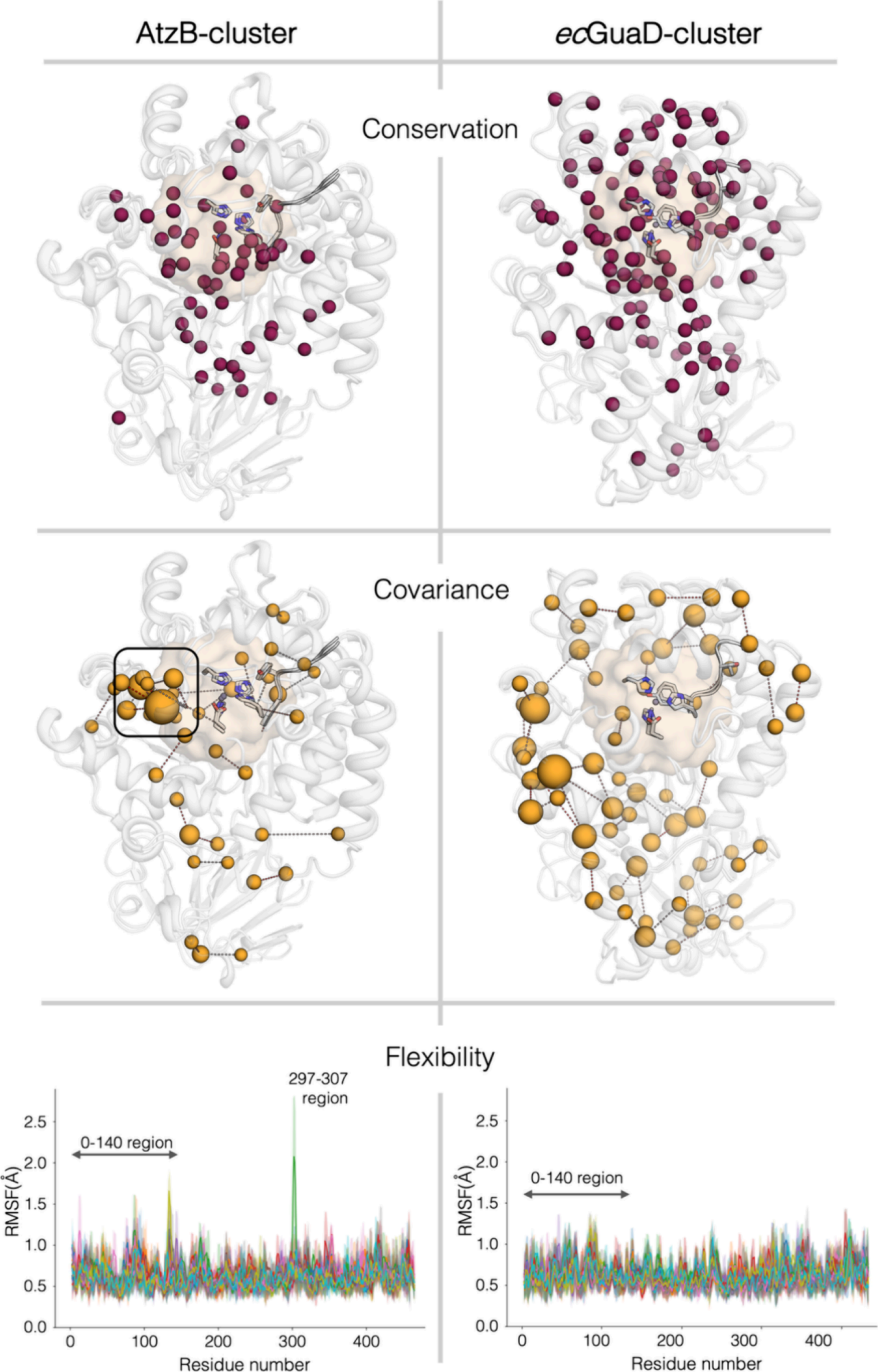
Comparative conservation, covariance, and flexibility analysis of the AtzB and *ec*GuaD clusters. The sequence conservation and sequence covariance (i.e., coevolution of residues) were extracted from the individual AtzB and *ec*GuaD cluster MSAs and mapped onto the 3D structures via ConSurf^44^ and VisualCMAT.^45^ Burgundy spheres indicate strong conservation, while orange spheres indicate strong covariance. The sphere radius in the middle panel is proportional to the sum of *z*-scores of the respective position with other coevolving positions, with larger sum of *z*-scores indicating higher covariance.^45^ A loop-helix region that shields the active site and shows a strong covariance in AtzB cluster enzymes is framed with a black box (297-307 region). The structures of *po*NdmH and *hs*NdmH as well as *po*GuaD and *hs*GuaD are exemplarily shown as representatives for the AtzB and the *ec*GuaD cluster, respectively. The beige-colored meshed surfaces include residues of 10 Å around the divalent metal centers, thereby indicating the respective active site pocket. The per-residue RMSF plots (in Å) are obtained from the five replica 100 ns MD simulations, each color representing a replicate and system (*po*NdmH and *hs*NdmH trajectories for the AtzB cluster, and *po*GuaD and *hs*GuaD simulations for the *ec*GuaD cluster). The most relevant differences lie in the 1–140 and 297–307 regions: 1–140 contains part of the (αβ)-domain, the region containing Y133, and the alpha Helix 1 (αH1), while the 297–307 region is located close to the loop containing the catalytically relevant E243 (cf. Figure S18).

Based on the clear differences in promiscuity and evolvability between the AtzB and *ec*GuaD clusters, we decided to computationally evaluate and compare the conformational dynamics of representative enzymes from each cluster. Specifically, we conducted Molecular Dynamics (MD) simulations of *hs*NdmH and *po*NdmH from the AtzB cluster, and *hs*GuaD and *po*GuaD included in the *ec*GuaD cluster. This allows for direct comparisons of NdmH and GuaD from the same organism, as these representatives stem from *Haliea sp*. SAOS-164 and *Pleomorphomonas oryzae*, respectively. Global flexibility analysis of these enzymes via Root Mean Square Fluctuation (RMSF) calculation indicates that *hs*NdmH and *po*NdmH are more flexible than the GuaD scaffolds, especially in the 1–140 and 297–307 regions (Figure 6, bottom panel). The 1–140 section contains part of the (αβ)-domain, the loop containing Y133, and α-helix 1 (αH1), while the 297–307 region is located close to the loop containing the catalytically relevant E243 (Figure S18).

From these MD simulations, we reconstructed conformational landscapes based on a key distance involving the closed-to-open conformational change of αH1­(*x*-axis) and different active site residues as described by principal component analysis (PCA, *y*-axis), which are shown in Figure 7A and Figure S19. Interestingly, *hs*NdmH and *po*NdmH adopt multiple conformational states presenting distances of the αH1 in the range of 17.5–23 Å, as well as a different level of closure of the active site pocket (*hs*NdmH explores four major conformations, whereas *po*NdmH adopts five major states). Such flexibility of the active site mostly involves the loop containing the catalytically relevant E243 (Figure 7B, named the E-region) and the region containing Y133 (Figure 7B, named the Y-region). Positive values of the active site PC indicate a shorter distance between the E- and Y-regions, thus indicating a more closed conformation of the E- and Y-loops (Figure 7A). Interestingly, a single mutation of E243 or Y133 to alanine severely impacts the enzyme catalytic activity (Figure 4B), either due to the direct involvement of the wild-type residue in catalysis (E243) or due to the close proximity to the substrate’s exocyclic oxygen (Y133), thereby likely aiding in the stabilization of the oxyanion intermediate. Altogether, these results indicate that a higher flexibility of both regions is beneficial for evolvability and substrate promiscuity, as it allows E243 and Y133 to adopt a catalytically relevant positioning. The conformational landscapes of *hs*GuaD and *po*GuaD indicate a rather low conformational flexibility, as mostly one major state is visited, presenting substantially smaller distances of αH1 (∼17 Å), thus indicating a more rigid and closed conformation of αH1. Similarly, E- and Y-regions display a restricted flexibility as compared to NdmH enzymes, as only intermediate active site PC values (around 0) are found for *hs*GuaD, and a slightly more open state is found for *po*GuaD (more negative (i.e., open) active site PC values). Along the AtzB to AtzB-CQNN trajectory,^9^ the conformation of αH1 containing the substrate-flanking residues W93 and Y98 (in *po*NdmH numbering) was found to be important for altering the enzyme specificity for guanine/hydroxyatrazine. Indeed, the new conformational landscapes reconstructed for NdmH and GuaD proteins confirm that the flexibility of αH1, together with the E- and Y-loop regions, is the most important conformational change dictating the promiscuity and evolvability of the enzyme scaffolds. These results are in line with promiscuity and evolvability being linked to the enzyme dynamical nature.^46^ Last, we evaluated the pathway of conformationally relevant positions via the shortest path map (SPM) analysis.^47,48^ This SPM analysis identifies a higher number of conformationally relevant residues for NdmH as compared to GuaD enzymes, which ranges from ca. 55 to 25, respectively (N value in Figure 7C). This more expanded conformational network found for *hs*NdmH and *po*NdmH (N equals 51 and 58, respectively) is again in line with a higher flexibility, underscoring their pronounced promiscuity and evolvability found experimentally (Figure 7C).

**Figure 7:**
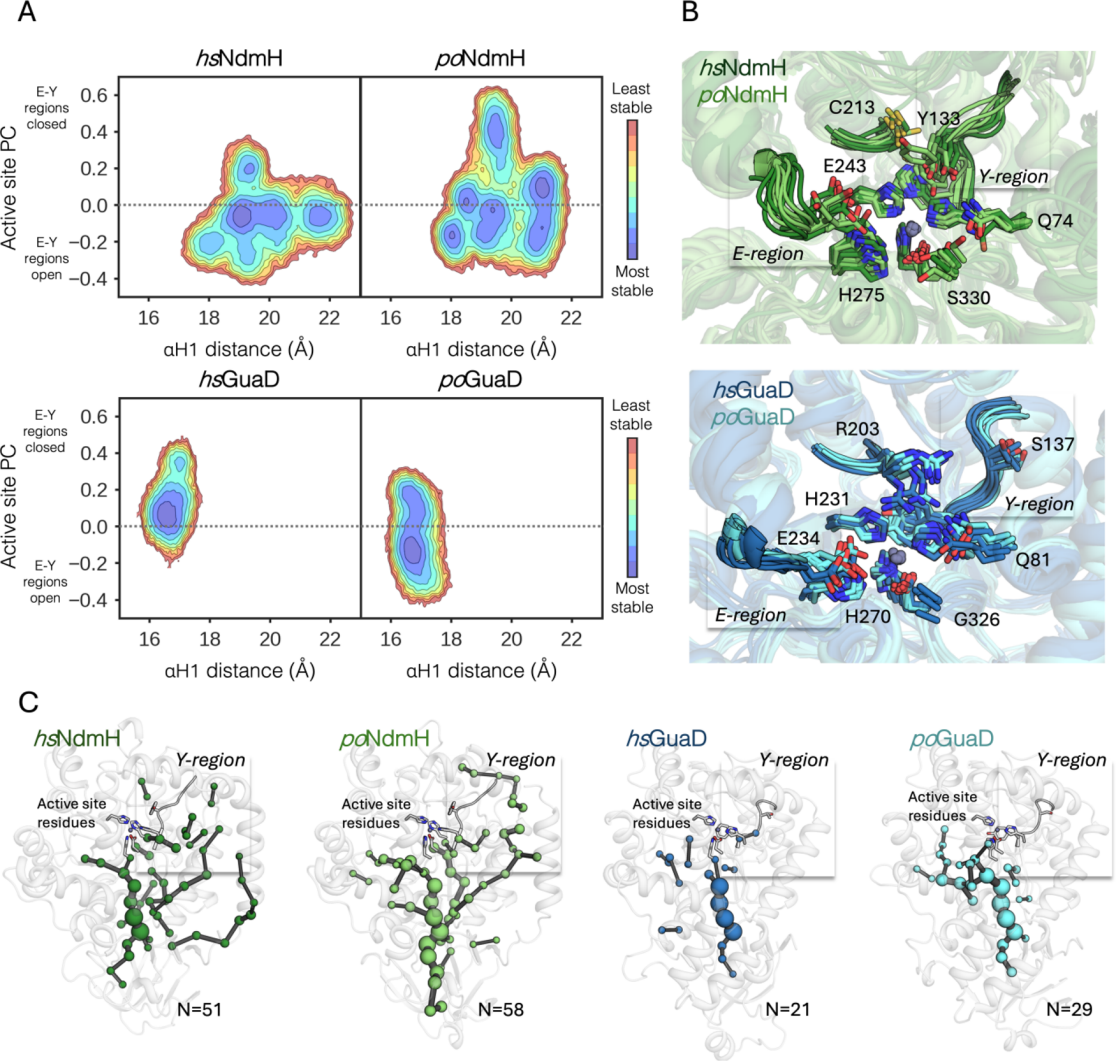
(A) Reconstructed conformational landscapes of *hs*NdmH, *po*NdmH, *hs*GuaD, and *po*GuaD in the apo state describing the α helix 1 opening distance (αH1, *x*-axis, cf. Figure S19 for more details) and the movement of the active site residues (PCA-generated *y*-axis considering distances between Cα atoms). Positive PC values correspond to shorter distances between the E- and Y-loops and thus indicate a more closed active site and a more closed E–Y conformation. A red-to-blue coloring scheme is used (red for the least populated conformation and blue for the most populated ones). (B) Overlay of representative structures extracted from the conformational landscapes. NdmH systems from the AtzB cluster (top panel, *hs*NdmH and *po*NdmH in dark green and light green, respectively) are more flexible, particularly in the region where the active site residues E243 and Y133 are located (named the E- and Y-regions, respectively). Conversely, GuaD systems from the *ec*GuaD cluster (bottom panel, *hs*GuaD and *po*GuaD in dark blue and light blue, respectively) show a more restricted conformational heterogeneity in those regions. For clarity, the numbering used is that of *po* systems (i.e., NdmH and GuaD from *Pleomorphomonas oryzae*). (C) Shortest path map (SPM) analysis of the studied systems. The SPM networks of NdmH systems capture conformationally relevant hotspots near position Y133, whereas GuaD systems do not (in line with the larger flexibility in the Y-region that NdmH systems show). The number of residues (*N*) contained in each SPM pathway is displayed.

## Conclusions

The study of enzymes degrading recently released anthropogenic substances and their evolution from mechanistically related precursors contributes to a better understanding of nature’s blueprint to generate novel enzymes. It is commonly accepted that promiscuous side activities are the starting points for the evolution of new enzymes and that flexibility and evolvability might be connected.^9,46,49,50^ However, it is not fully understood which features make an enzyme promiscuous and evolvable. To investigate this issue, we have previously shown that the xenobiotic-degrading enzyme hydroxyatrazine ethylaminohydrolase AtzB shows promiscuous guanine deaminase activity and have experimentally retraced an evolutionary path from a GuaD-precursor to AtzB.^9^ Moreover, we have recently provided mechanistic evidence that GuaD, AtzB, and homologues catalyze hydrolysis reactions via a 1,6 nucleophilic conjugate addition and that this mechanistic commonality has facilitated the evolution of AtzB, as guanine and hydroxyatrazine share the conserved motif required for productive catalysis (Figure 1A,C, indicated in red).^24^ In the present work, we have characterized a hitherto unknown enzymatic reaction, *N* ^2^,*N* ^2^-dimethylguanine hydrolysis, elucidated the underlying catalytic mechanism, assigned this function as native to the AtzB homologues (NdmHs), and provided evidence that AtzB has evolved from an enzyme with this function. Although mechanistically not different from GuaD enzymes, these newly identified NdmH enzymes exhibit superior substrate promiscuity and evolvability. Given the proposed connection between conformational dynamics and the promiscuity and evolvability of enzyme scaffolds,^46^ and considering that both enzyme classes operate through the same catalytic mechanism, we performed molecular dynamics (MD) simulations instead of complementary QM/MM mechanistic analyses. The computational evaluation of conservation, covariance, and flexibility studies, as well as conformational landscape reconstruction and correlation-based shortest path map (SPM) analysis showed that a higher conformational heterogeneity of the active site and a more flexible protein scaffold allow for these molecular properties. These findings are in accordance with the ensemble model of enzyme promiscuity, which posits that enzymes exist as dynamic ensembles of conformations, in which different conformations mediate different functions.^46^ Specifically, the observed flexibility of the E- and Y-region in NdmH enzymes (Figure 7) is beneficial, as it enables E243 and Y133 to adopt a catalytically relevant conformation. These E- and Y-regions of the active site are of particular importance for catalysis, as they stabilize the positive and negative poles of the elevated dipole moment of the tetrahedral intermediate, respectively, and thereby enable electric field catalysis (Figures S16 and S17). Thus, this flexibility likely allows for catalytically competent positioning of diverse substrates within the active site, similar to what we discuss for the changes in substrate specificity within the AtzB evolutionary trajectory (Figure 5D and Figure S14). Hence, our combined experimental and computational analysis of NdmH enzymes explains the rapid functional diversification in response to the release of anthropogenic substances into the environment and contributes to a better understanding of enzyme promiscuity and evolvability. In continuation of this work, we are currently investigating the physiological context of this new class of NdmH enzymes.

## Material and Methods

### Bacterial Strains and Chemicals

BL21 (DE3) Gold (Agilent Technologies) and NEB Turbo (New England Biolabs) *E. coli* strains were used for protein production and plasmid amplification, respectively. Chemicals were purchased from commercial sources and were of analytical grade or higher.

### Gene Cloning and Site-Directed Mutagenesis

Genes encoding wild-type proteins were codon-optimized for recombinant expression in *E. coli* and purchased from GeneArt (Thermo Fisher Scientific) as gene strings. Flanking *Bsa*I restriction sites enabled cloning of the genes into pUR22_*Bsa*I and pUR23_*Bsa*I expression vectors using a coupled digestion/ligation reaction with *Bsa*I and T4-DNA ligase.^51^ All protein variants were created by site-directed mutagenesis, employing a modified version of the QuikChange protocol. Primers harboring specific mismatches were designed to amplify the entire plasmid. The resulting linear amplicon was treated with T4 polynucleotide kinase and T4-DNA ligase (5 U; 10 U; 30 min at 37 °C; 30 min at 25 °C) in 1 × T4 ligase buffer in a total volume of 50 μL. The resulting ligation solution, containing circular, mutated plasmids, was used for the transformation of *E. coli* cells (NEB Turbo) without further purification. After plasmid isolation, the integrity of the construct and the presence of the desired mutation were confirmed in each case by Sanger sequencing (Microsynth Seqlab).

### Gene Expression and Protein Purification

All proteins analyzed in this study were produced as either N-terminal or C-terminal His_6_-tagged fusion constructs. Production of wild-type proteins and AtzB variants was reported previously,^9,24^ while production of all other variants is shown in the present study. For gene expression, the *E. coli* strain BL21 (DE3) Gold (Agilent Technologies) was transformed with the respective expression plasmid coding for the protein of interest. Cells were grown in LB medium (37 °C, 140 rpm) supplemented with 150 mg/mL ampicillin to an OD_600_ of 0.6, followed by induction of gene expression by the addition of 0.5 mM IPTG. Cells were cultivated overnight (20 °C, 16 h), harvested by centrifugation (4000*g*, 20 min, 4 °C), and resuspended in 100 mM Tris/HCl (pH 7.5), 300 mM KCl, and 10 mM imidazole. Afterward, cells were disrupted by sonication (Branson Sonifier W-250D, 60% amplitude, 3 min, 2 s pulse, 2 s pause) and cell debris and insoluble aggregates were removed by centrifugation (16,000*g*, 45 min, 4 °C). The proteins were purified from the supernatant by immobilized metal affinity chromatography (IMAC) using an ÄKTA-purifier system with a HisTrap excel column (GE Healthcare), applying a linear imidazole gradient (10 mM–1 M over 15 CV). The proteins were further purified by preparative size-exclusion chromatography (SEC) using an ÄKTA-purifier system with a HiLoad 26/60 Superdex 75 or a HiLoad 26/60 Superdex 200 column (GE Healthcare) equilibrated with 50 mM Tris/HCl (pH 7.5), 50 mM KCl. Protein elution was continuously monitored at 280 nm, and fractions containing the protein of interest were identified by SDS-PAGE and pooled. Protein concentrations were determined by absorption spectroscopy at 280 nm (Thermo Fisher Scientific, NanoDrop One) using a molar extinction coefficient that was calculated from the amino acid sequence.^52^ The purified proteins were frozen in liquid nitrogen and stored at −70 °C.

### HPLC-Based Enzyme Assays

To confirm the activity of an enzyme toward a certain substrate and to identify product composition, qualitative enzyme assays based on HPLC analysis were conducted. All enzymatic assays were performed in 50 mM potassium phosphate (pH 7.5) containing 500 μM of the respective substrate and 2 μM of the tested enzyme in a total volume of 150 μL. As a reference, genuine substance standards were set up in the same way without any enzyme present. Following incubation at 25 °C and 500 rpm for 24 h, all reactions were stopped by centrifugation using a filter tube with a pore size of 10 kDa to remove the enzymes. The reaction products were subsequently analyzed by reversed-phase HPLC using an Agilent system (1100 series) with an Eclipse XDB-C18 (4.6 × 150 mm) column. The separation was performed at 20 °C using 0.1% formic acid in water as buffer A and 0.1% formic acid in acetonitrile as buffer B with an isocratic elution (5% buffer B, 1 mL/min, 0–7.5 min) followed by a gradient elution (5–100% buffer B, 1 mL/min, 7.5–20 min).

### Steady-State Enzyme Kinetics

The difference in absorbance between *N* ^2^,*N* ^2^-dimethylguanine and xanthine at 294 nm (Δε_294_ = 3187 M^–1^cm^–1^), guanine and xanthine at 253 nm (Δε_253_ = 5023 M^–1^cm^–1^),^9^ and hydroxyatrazine and *N*-isopropylammelide at 242 nm (Δε_242_ = 10,957 M^–1^cm^–1^)^9^ was used to monitor the respective reaction with a spectrophotometer (JASCO V-750) via a direct photometric assay. These differential molar extinction coefficients were deduced from difference spectra of substance standards and were determined either in our present study (cf. Figure S4) or in our previous study.^9^ Catalytic parameters for the hydrolysis of *N* ^2^,*N* ^2^-dimethylguanine, guanine, and hydroxyatrazine were determined under steady-state conditions. Reactions were performed in triplicate at 25 °C. Standard assays contained varying amounts of *N* ^2^,*N* ^2^-dimethylguanine, guanine, and hydroxyatrazine, respectively, in a total volume of 300 μL of 50 potassium mM phosphate buffer (pH 7.5). When a constant baseline absorbance was reached, reactions were initiated by the addition of the respective enzyme. In all cases, it was assured that the enzyme concentration was at least 10 times lower than the substrate concentration. Initial velocities (*v*_i_) were calculated from the initial linear part of the resulting curve via division by the respective differential molar extinction coefficient. The determined reaction velocities were then normalized to the applied enzyme concentration (*v*_i_/[*E*_0_]) and plotted against the substrate concentration. By fitting the data to the Michaelis–Menten equation in Origin 2022 (OriginLab Corporation), the Michaelis constant *K*_M_ and turnover number *k*_cat_ were determined.

### Time-Dependent UV-Based Activity Measurements

To monitor the progress of enzymatic *N* ^2^,*N* ^2^-dimethylguanine hydrolysis and to analyze product composition, absorbance spectra of 1 mM *N* ^2^,*N* ^2^-dimethylguanine in 50 mM potassium phosphate (pH 7.5) were recorded between 200 and 320 nm with a spectrophotometer (JASCO V-750). Then, 200 nM *po*NdmH was added to the reaction mixture and absorbance spectra were recorded every 10–50 s. After 400 s, the absorbance spectrum was equivalent to the absorbance spectrum of a xanthine standard. Further incubation of the reaction mixture for 3600 s did not result in further changes of the absorbance spectra.

### Temperature-Dependent UV-Based Activity Measurements

Unless stated otherwise, activity measurements were set up and conducted in the same way as described in the section “Steady-State Enzyme Kinetics”. Activities of AtzB and AtzB-CQNN toward *N* ^2^,*N* ^2^-dimethylguanine and hydroxyatrazine were measured in triplicates at 278, 283, 288, 293, 298, and 303 K. The experimental conditions included 50 mM potassium phosphate (pH 7.5) and 2000 μM *N* ^2^,*N* ^2^-dimethylguanine or 100 μM hydroxyatrazine, respectively. In all cases, it was assured that the enzyme concentration was at least 20 times lower than the substrate concentration and that identical concentrations of enzyme were used for the measurements at different temperatures. Then, for each temperature, *k*_cat_ was approximated using the Michaelis–Menten equation, values for *K*_M_ as determined by steady-state enzyme kinetics, and applied substrate concentrations. Values for the activation energy (*E*_a_), the enthalpy of activation (Δ*H* ^⧧^), the entropy of activation (Δ*S* ^⧧^), and the Gibbs energy of activation at 298 K (Δ*G* ^⧧ (298)^) were obtained by fitting the data to the linear Arrhenius and Eyring equation (cf. Figure S14) via Origin 2022 (OriginLab Corporation). *E*_a_ was calculated from the slope of the Arrhenius plot, while Δ*H* ^⧧^ was calculated from the slope of the Eyring plot. Δ*S* ^⧧^ was calculated from the y-intercept of the Eyring plot using a value of 0.8 for κ, typically applied for enzymatic reactions.^31,38,53^ T*Δ*S* ^⧧ (298)^ was calculated as 298 K*Δ*S* ^⧧^, while Δ*G* ^⧧ (298)^ was calculated as Δ*H* ^⧧^-298 K*Δ*S* ^⧧ (298)^ (cf. Figure 5D). Last, the respective difference for each thermodynamic parameter was determined for the evolutionary process AtzB-CQNN → AtzB (e.g.: ΔΔ*H* ^⧧^ = Δ*H* ^⧧^_AtzB‑CQNN_ – Δ*H* ^⧧^_AtzB_).

### Analytical Size-Exclusion Chromatography

Analytical size-exclusion chromatography was performed using a Superdex S200 increase 10/300 GL column (GE Healthcare) operated on an KTAmicro system (GE Healthcare). After equilibration with running buffer (50 mM Tris/HCl pH 7.5, 50 mM KCl), 50 μM of the respective protein was applied to the column. Protein elution was performed at 25 °C with a flow rate of 0.3 mL/min and was monitored by absorbance measurements at 280 nm. Calibration was performed with the Cytiva LMW and HMW calibration kits. The oligomerization state was calculated by using the apparent molecular weight and the expected molecular weight of the respective monomer.

### Circular Dichroism (CD) Spectroscopy

Far-UV CD spectroscopy was used to assess the structural integrity of the *po*NdmH alanine mutants. Spectra were recorded with a CD spectrometer (J-815, JASCO) between 195 and 250 nm using a quartz cuvette (0.2 mm). Measurements were conducted in five replicas at 25 °C using 10 μM of the respective protein in 50 mM Tris/HCl (pH 7.5) and 50 mM KCl. All spectra were corrected for buffer absorption and smoothed using the Savitzky–Golay algorithm (convolution width 9) implemented in the Spectra Analysis software provided by JASCO. The mean molar ellipticity per-residue θ [deg cm^2^ dmol^–1^] was calculated from the observed ellipticity θ_obs_ [mdeg], the path length of the cuvette *d* [cm], the protein concentration *c* [μM], and the number of peptide bonds per protein *N*_A_ via the following equation:

$$\theta =\frac{\theta_{\mathrm{obs}}\cdot 10^{5}}{c\cdot d\cdot N_{A}}$$

### Sequence Similarity Networks (SSN)

To generate sequence similarity networks, homologous sequences of previously characterized AHS enzymes (AtzB, *po*NdmH, *hs*NdmH, *rb*NdmH, *pa*8-OxoGuaD, *ec*GuaD, *po*GuaD, and *hs*GuaD)^24^ were retrieved by BLAST searches using standard parameters (5000 hits).^54^ Then, the sequences were submitted to the EFI-EST Web server and SSNs were created to cluster closely related sequences using standard parameters.^55^ SSNs with a 90% representative node identity (RepNode90) were visualized in Cytoscape (3.10.1) at a sequence identity threshold of 35.0%.

### Multiple Sequence Alignment (MSA)

Two individual MSAs for both the AtzB cluster and the *ec*GuaD cluster of the SSN were generated with MAFFT,^56^ and the sequence conservation of selected positions was analyzed via JalView.

### Mapping Sequence Conservation and Sequence Covariance onto Protein Structures

To structurally analyze sequence variation within a protein scaffold, the sequence conservation and the sequence covariance (i.e., coevolving residues) were deduced from an MSA and mapped onto protein 3D structures via ConSurf^44^ and VisualCMAT,^45^ respectively. Specifically, the individual MSAs constructed for the AtzB cluster and the *ec*GuaD cluster together with representative structures of each cluster (AtzB cluster: *po*NdmH AlphaFold model; *ec*GuaD cluster: *ec*GuaD PDB crystal structure 6OHB) were submitted to both Web servers, and mapping of conservation and covariance, respectively, was conducted with standard parameters.

### Analysis of Protein Structures

Crystal structures are available for *ec*GuaD (PDB: 6OHB, 6OHC). For all other experimentally analyzed enzymes, structural models were generated with AlphaFold3.^57^ Protein structures were analyzed and visualized in PyMol.

### Estimation of Dipole Moments

To compare the dipole moments of the substrate *N* ^2^,*N* ^2^-dimethylguanine (cf. Figure 4C-I), the tetrahedral intermediate of *N* ^2^,*N* ^2^-dimethylguanine hydrolysis (cf. Figure 4C-III), the substrate hydroxyatrazine (cf. Figure S15I), and the tetrahedral intermediate of hydroxyatrazine hydrolysis (cf. Figure S15III), we examined the isolated structures and conducted an energy-minimization step for each of the four molecules individually by employing the MM2 force field implemented in Chem3D using standard parameters. Then, the dipole moments of these energy-minimized conformations were estimated and visualized in Avogadro (cf. Figure S16).^58^

### Molecular Modeling System Preparation

The starting structures for the four systems (*hs*NdmH, *po*NdmH, *hs*GuaD, and *po*GuaD) were generated using the AlphaFold3 (AF3)^57^ neural network, all as homodimers with a Zn­(II) ion placed in each active site. The AF3 models had a predicted template modeling (pTM) score higher than 0.94. The metal ion binding site parameters were generated using the MCPB.py program in the Amber22 software package,^59^ which consists of a Zn­(II) ion penta-coordinated by three histidine residues, an aspartate residue, and a water molecule. The partial charges and bonded parameters for metal-binding site residues were generated with the Gaussian09 software package by utilizing the B3LYP/6-31G* level of theory. The crystallographic water molecules of *ec*GuaD (PDB: 6OHC) were added to each homodimer. The protonation states were predicted using PROPKA.^60,61^ A total of five replicas of MD simulations at the NVT ensemble in the absence of any ligand were performed reaching a total simulation time of 0.5 μs/system (5 replicas × 100 ns) for *hs*NdmH, *po*NdmH, *hs*GuaD, and *po*GuaD. The MD simulation protocol and details used are explained in the Supporting Information and elsewhere.^62,63^

### Conformational Landscape Reconstruction

Molecular dynamics (MD) simulations allow the sampling of the population distribution of biomolecules by integrating Newton’s laws of motion. This probability distribution of molecular states is represented in an extremely high-dimensional space. This is usually solved by focusing on a selected set of degrees of freedom (DOF) that is relevant to the process of interest. In our case, we used the distance between His69 and Val90 (*po*NdmH numbering as reference) for the closed-to-open transition of the α helix 1 (αH1, *x*-axis, cf. Figure S19 for more details) and Principal Component 1 based on the PCA analysis considering distances between Cα atoms of the active site residues to describe the open-to-closed transition of the active site (*y*-axis).

### Shortest Path Map (SPM) Calculations

The shortest path map (SPM) analysis was performed by using the MD simulations of all analyzed systems. For SPM calculation, the MD simulations are used to compute the interresidue mean distance and correlation matrices, which are then used for graph construction and analysis. All SPM analyses performed used a distance threshold of 6 Å, and a significance threshold of 0.3. More details about our SPM tool can be found elsewhere.^47,48^

## Supplementary Material



## Notes

Sequence similarity network and sequence comparisons; steady-state enzyme kinetics of *po*GuaD, *hs*GuaD, and *rb*NdmH with guanine as a substrate; screened substrates and catalyzed reactions; determination of the differential molar extinction coefficient (Δε) at 294 nm for *N* ^2^,*N* ^2^-dimethylguanine and xanthine; steady-state enzyme kinetics of AtzB, *po*NdmH, *hs*NdmH, *rb*NdmH, and AtzB-CQNN with *N* ^2^,*N* ^2^-dimethylguanine as a substrate; analytical size-exclusion chromatography of AtzB and NdmHs; assessment of the purity of *po*NdmH alanine mutants; assessment of the secondary structure of purified *po*NdmH alanine mutants by far-UV circular dichroism (CD) spectroscopy; steady-state enzyme kinetics of *po*NdmH alanine mutants with *N* ^2^,*N* ^2^-dimethylguanine as a substrate; metal-binding motif of AHS subtype III members; steady-state enzyme kinetics of previously generated AtzB mutants with *N* ^2^,*N* ^2^-dimethylguanine as a substrate; assessment of the purity of NdmH-SSII mutants; steady-state enzyme kinetics of several NdmH variants with *N* ^2^,*N* ^2^-dimethylguanine and hydroxyatrazine as substrates; temperature-dependent activity measurements (278–303 K) for the hydrolysis of hydroxyatrazine and *N* ^2^,*N* ^2^-dimethylguanine by wild-type AtzB and AtzB-CQNN; reaction mechanism for the hydrolysis of hydroxyatrazine; estimation of dipole moments; reaction scheme for electric field catalysis; structural superposition of *po*NdmH and *po*GuaD; key structural regions of the conformational landscape analysis; MD simulation details; catalytic efficiencies *k*_cat_/*K*_M_ determined under steady-state kinetic conditions; characteristics and amino acid sequences of the analyzed AHS enzymes; and supporting references (PDF)
