# Supplementary material for: The Discovery of N 2,N 2‑Dimethylguanine Hydrolases Unravels General Molecular Principles of Enzyme Evolvability and Promiscuity
Source: ACS Catal. 2026 Mar 23;16(7):6865–79. doi: 10.1021/acscatal.6c00436 (PMC13054789; doi:10.1021/acscatal.6c00436)
Supplement: Supplementary file 1 [file cs6c00436_si_001.pdf]

Supporting Information

The discovery of N^2,N^2-dimethylguanine hydrolases unravels general molecular principles of enzyme evolvability and promiscuity

Lukas Drexler[1], Cristina Duran[2], Sílvia Osuna[2,3], Reinhard Sterner[1]**

[1]Institute of Biophysics and Physical Biochemistry, Regensburg Center for Biochemistry, University of Regensburg, D-93040 Regensburg, Germany.

[2]Institut de Química Computacional i Catàlisi(IQCC) and Departament de Química,Universitat de Girona, Girona 17003, Spain

[3]ICREA, Barcelona 08010, Spain

***Corresponding Author:**
Sílvia Osuna: Email: silivia.osuna@udg.edu
Reinhard Sterner: Email: reinhard.sterner@ur.de

ORCID

Lukas Drexler: orcid.org/0000-0001-8193-3256

Cristina Duran: orcid.org/0000-0003-3094-8823

Sílvia Osuna: orcid.org/0000-0003-3657-6469

Reinhard Sterner: orcid.org/0000-0001-8177-8460

A

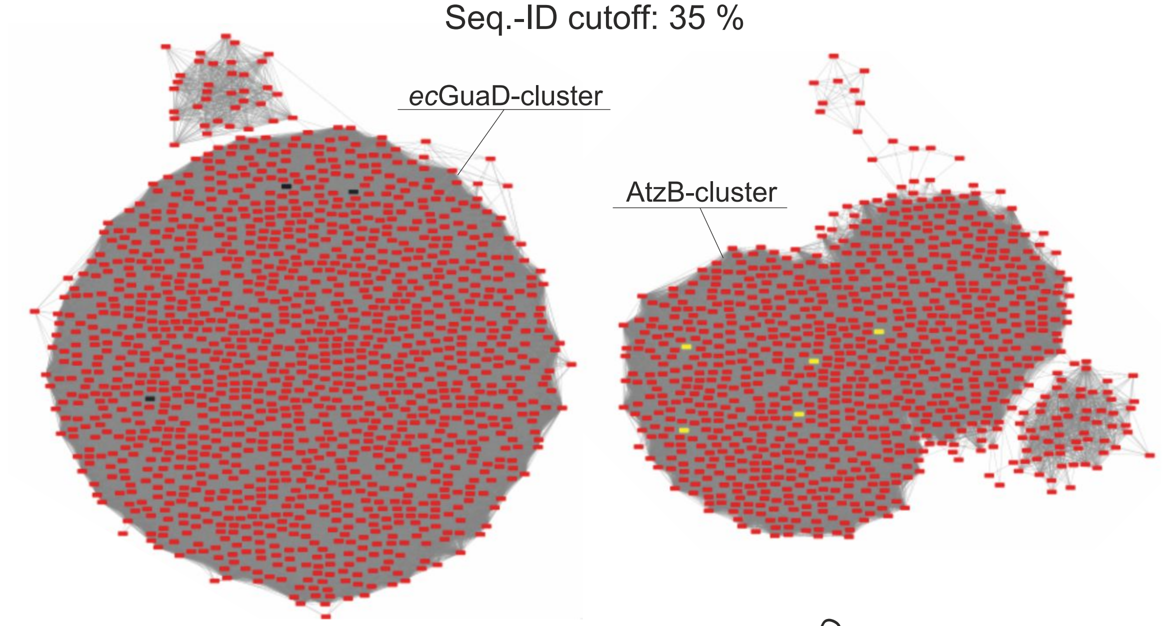

B

	Seq.-ID [%]	*AtzB*	*poNdmH*	*hsNdmH*	*rbNdmH*	*pa8-OxoGuaD*	*ecGuaD*	*poGuaD*	*hsGuaD*
yellow	AtzB	100	60	65	58	36	25	26	27
yellow	*po*NdmH	60	100	57	78	35	25	26	26
yellow	*hs*NdmH	65	57	100	59	40	28	26	28
yellow	*rb*NdmH	58	78	59	100	36	24	24	26
yellow	*pa*8-OxoGuaD	36	35	40	36	100	28	28	29
black	*ec*GuaD	25	25	28	24	28	100	44	45
black	*po*GuaD	26	26	26	24	28	44	100	45
black	*hs*GuaD	27	26	28	26	29	45	45	100

Figure S1: (**A**) A sequence similarity network of 11000 unique sequences of guanine deaminases[1] was generated using EFI-EST[2] and visualized at a sequence identity (Seq.-ID) cutoff of 35.0 %. At this threshold, a cluster harboring AtzB, *po*NdmH, *hs*NdmH, *rb*NdmH, and *pa*8-OxoGuaD (marked by yellow rectangles) separates from the cluster harboring *ec*GuaD, *po*GuaD, and *hs*GuaD (marked by black rectangles). (**B**) Pairwise sequence identities between these previously characterized guanine deaminases.[1]

*po*GuaD + Guanine

k_{cat} = 55.6 s^{-1}

K_M = 23.4 µM

k_{cat}/K_M = 2.4 * 10^6 $M^{-1}s^{-1}$

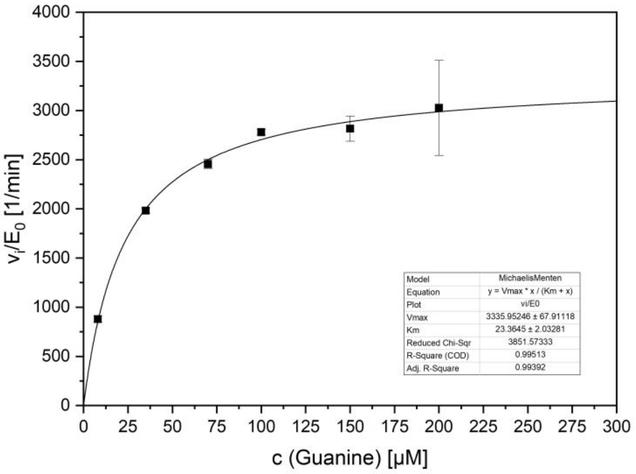

---

*hs*GuaD + Guanine

k_{cat} = 23.3 s^{-1}

K_M = 14.6 µM

k_{cat}/K_M = 1.6 * 10^6 $M^{-1}s^{-1}$

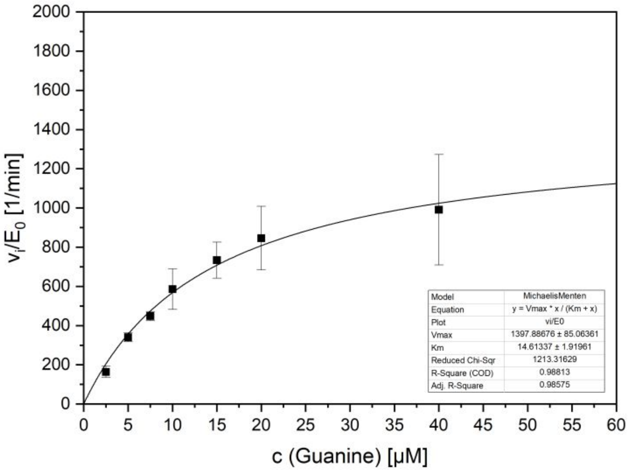

---

*rb*NdmH + Guanine

k_{cat} = 0.0855 s^{-1}

K_M = 326 µM

k_{cat}/K_M = 2.6 * 10^2 $M^{-1}s^{-1}$

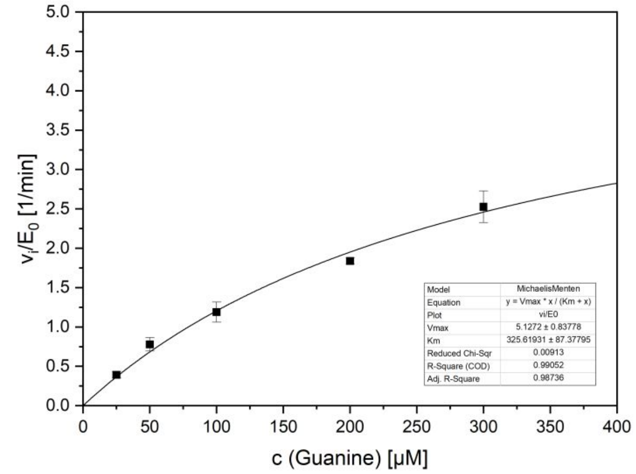

Figure S2: Steady-state enzyme kinetics of *po*GuaD, *hs*GuaD, and *rb*NdmH with guanine as substrate. The experimental conditions included 50 mM potassium phosphate (pH 7.5) and varying concentrations of guanine. The Michaelis constant K_M and the turnover number k_{cat} were obtained by fitting the data from triplicate measurements at 25 °C to the Michaelis-Menten equation using Origin 2022 (© OriginLab Corporation). Catalytic efficiencies (k_{cat}/K_M) of all enzymes are listed in Figure 2A, together with previously determined catalytic efficiencies for AtzB[3], *po*NdmH[3], *hs*NdmH[3], *pa*8-OxoGuaD[4], and *ec*GuaD[5].

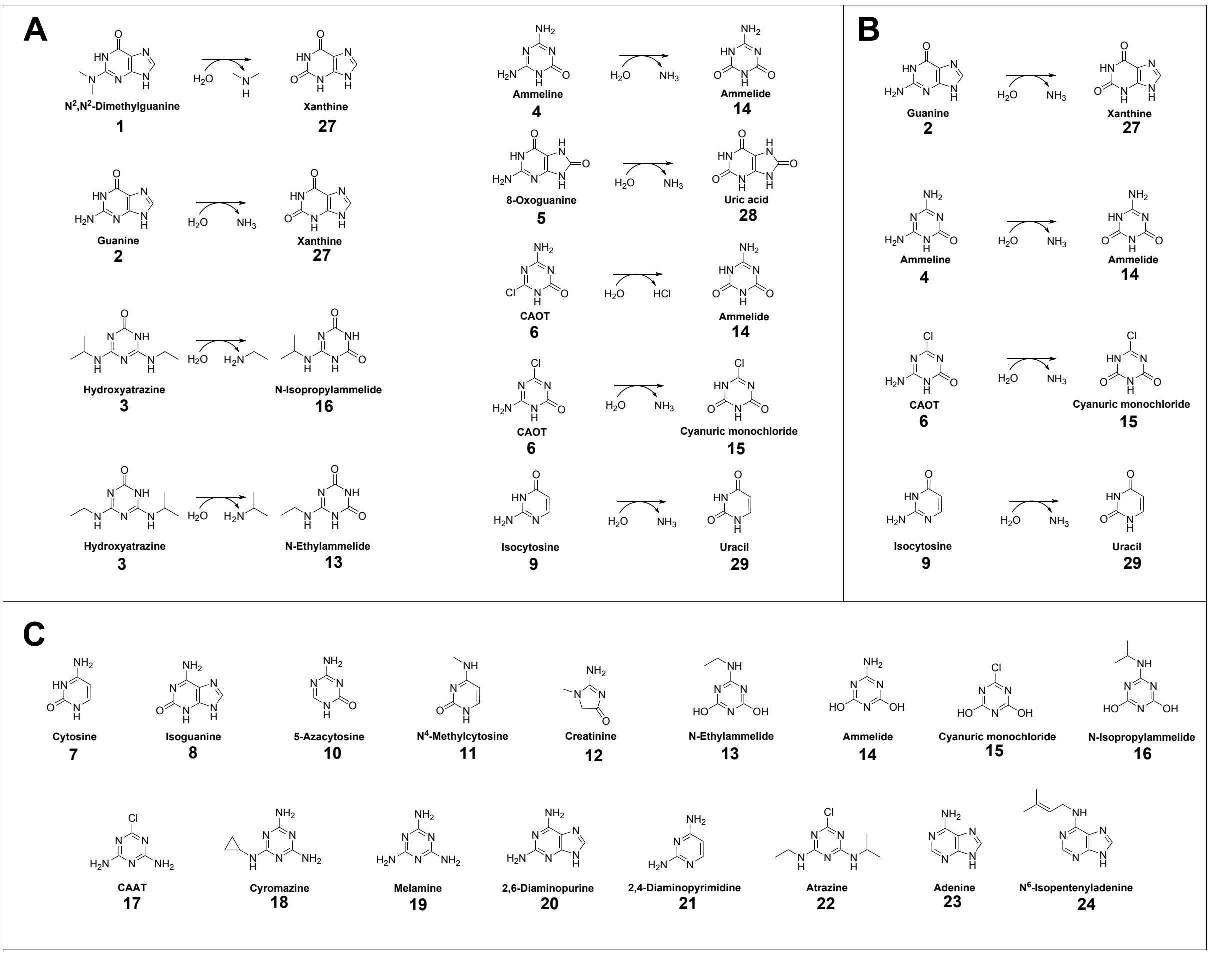

Figure S3: Substrate profiling using HPLC-based enzymatic assays revealed (**A**) nine different hydrolysis reactions for AtzB-cluster enzymes and (**B**) four different hydrolysis reactions for *ec*GuaD-cluster enzymes. (**C**) Compounds **7, 8,** and **10-24** were not hydrolyzed by any of the enzymes tested.[1] No activity was detectable towards the nucleoside N^2,N^2-dimethylguanosine.

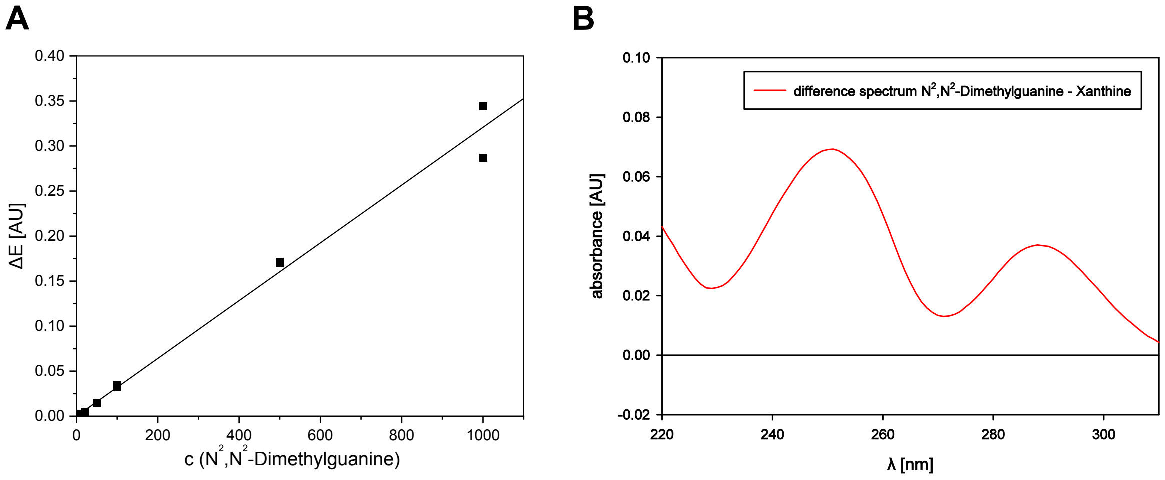

$\Delta\varepsilon$(294 nm) = 3187 [M^{-1} cm^{-1}]

Figure S4: Determination of the differential molar extinction coefficient (Δε) at 294 nm for N^2,N^2-dimethylguanine and xanthine. A value of 3187 [M^{-1} cm^{-1}] for Δε(294 nm) was determined by two approaches: (**A**) The maximum absorbance changes (*i.e.*, total substrate turnover) at 294 nm (ΔE) upon addition of 10 nM *po*NdmH to defined concentrations of N^2,N^2-dimethylguanine in 50 mM potassium phosphate (pH 7.5) allowed to plot the depicted calibration curve. Δε(294 nm) was calculated from the slope of this curve using Lambert-Beer's law. (**B**) UV absorption spectra of 100 µM N^2,N^2-dimethylguanine and xanthine, respectively, in 50 mM potassium phosphate (pH 7.5) were recorded by triplicate measurements. The spectrum of xanthine was subtracted from the spectrum of N^2,N^2-dimethylguanine resulting in the depicted difference spectrum, which was also used to calculate the Δε(294 nm) via Lambert-Beer's law.

AtzB + N^2,N^2-Dimethylguanine

k_{cat} = 4.2 s^{-1}

K_M = 5123 µM

k_{cat}/K_M = 8.2 * 10^2 $M^{-1}s^{-1}$

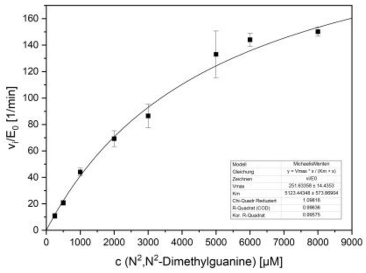

*po*NdmH+ N^2,N^2-Dimethylguanine

k_{cat} = 16.9 s^{-1}

K_M = 31.5 µM

k_{cat}/K_M = 5.4 * 10^5 $M^{-1}s^{-1}$

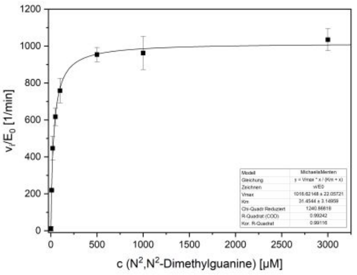

*hs*NdmH+ N^2,N^2-Dimethylguanine

k_{cat} = 26.4 s^{-1}

K_M = 159 µM

k_{cat}/K_M = 1.7 * 10^5 $M^{-1}s^{-1}$

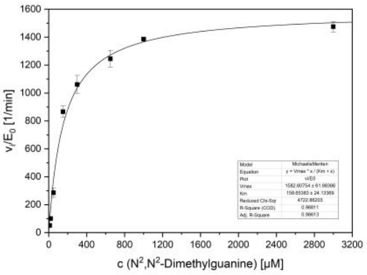

*rb*NdmH + N^2,N^2-Dimethylguanine

k_{cat} = 10.4 s^{-1}

K_M = 11.1 µM

k_{cat}/K_M = 9.4 * 10^5 $M^{-1}s^{-1}$

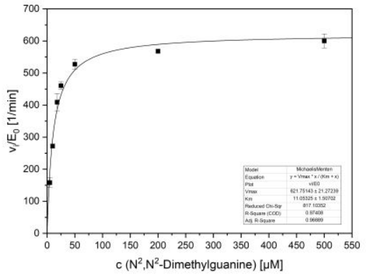

AtzB-CQNN + N^2,N^2-Dimethylguanine

k_{cat} = 12.4 s^{-1}

K_M = 52.5 µM

k_{cat}/K_M = 2.4 * 10^5 $M^{-1}s^{-1}$

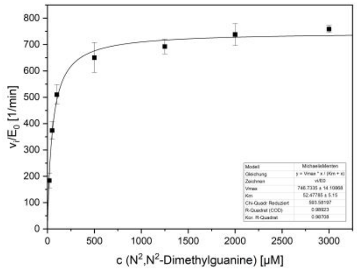

Figure S5: Steady-state enzyme kinetics of AtzB, *po*NdmH, *hs*NdmH, *rb*NdmH, and AtzB-CQNN with N^2,N^2-dimethylguanine as substrate. The experimental conditions included 50 mM potassium phosphate (pH 7.5) and varying concentrations of N^2,N^2-dimethylguanine. The Michaelis constant K_M and the turnover number k_{cat} were obtained by fitting the data from triplicate measurements at 25 °C to the Michaelis-Menten equation using Origin 2022 (© OriginLab Corporation). Catalytic efficiencies (k_{cat}/K_M) of all enzymes are listed in Figure 3D.

A

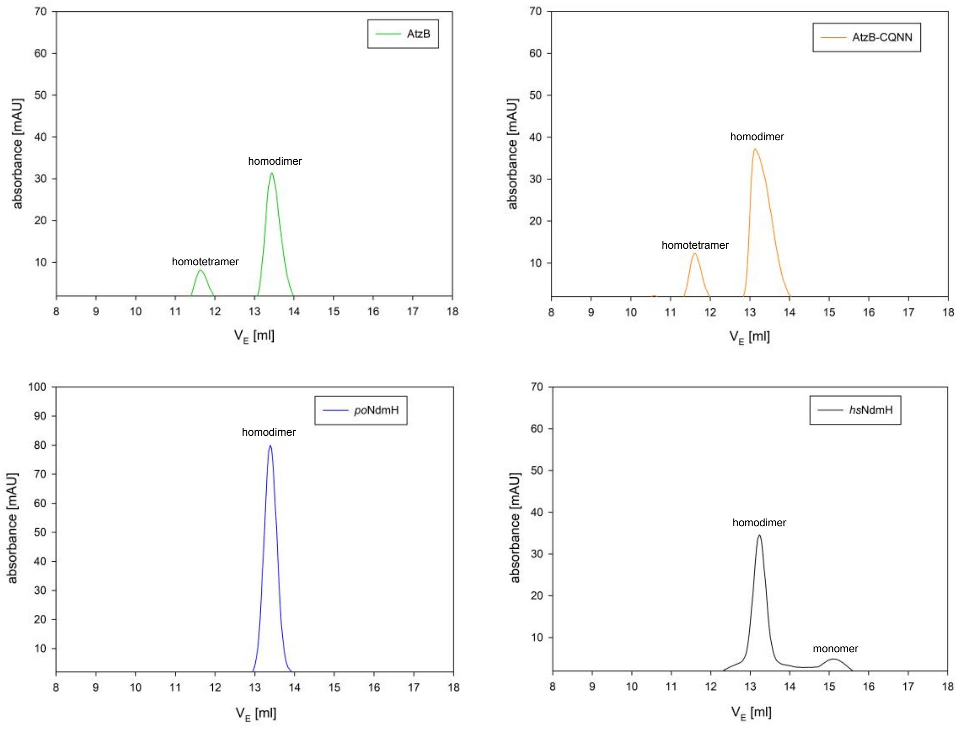

B

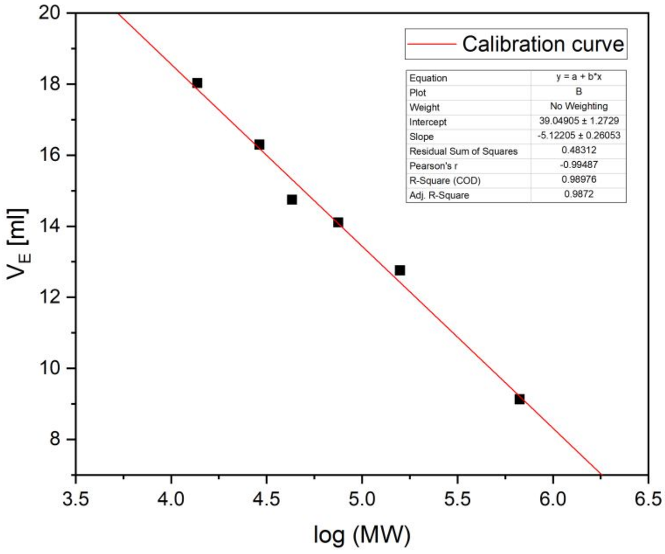

C

Enzymes	Oligomer	MW (theoretical) [kDa]	Elution volume [ml]	MW (apparent) [kDa]
AtzB	homodimer	106.4	13.4	99.9
	homotetramer	212.8	11.6	225.5
AtzB-CQNN	homodimer	106.4	13.1	114.9
	homotetramer	212.8	11.6	227.5
*po*NdmH	homodimer	102.4	13.4	102.2
*hs*NdmH	monomer	50.7	15.1	47.2
	homodimer	101.4	13.2	109.8

Figure S6: (**A**) Analytical size-exclusion chromatography (Superdex S200 column) at 25 °C with 50 µM of the indicated protein using 50 mM Tris/HCl (pH 7.5), 50 mM KCl as running buffer. The elution volumes V_E were determined by measuring protein absorbance at 280 nm. (**B**) The elution volumes of standard proteins with known molecular weight (MW) were used to generate a calibration curve. (**C**) Apparent molecular weights and oligomerization states were calculated using the observed elution volumes and the calibration curve. AtzB, AtzB-CQNN, *po*NdmH, and *hs*NdmH showed main peaks at elution volumes that correspond to the expected molecular weight of the respective homodimer.

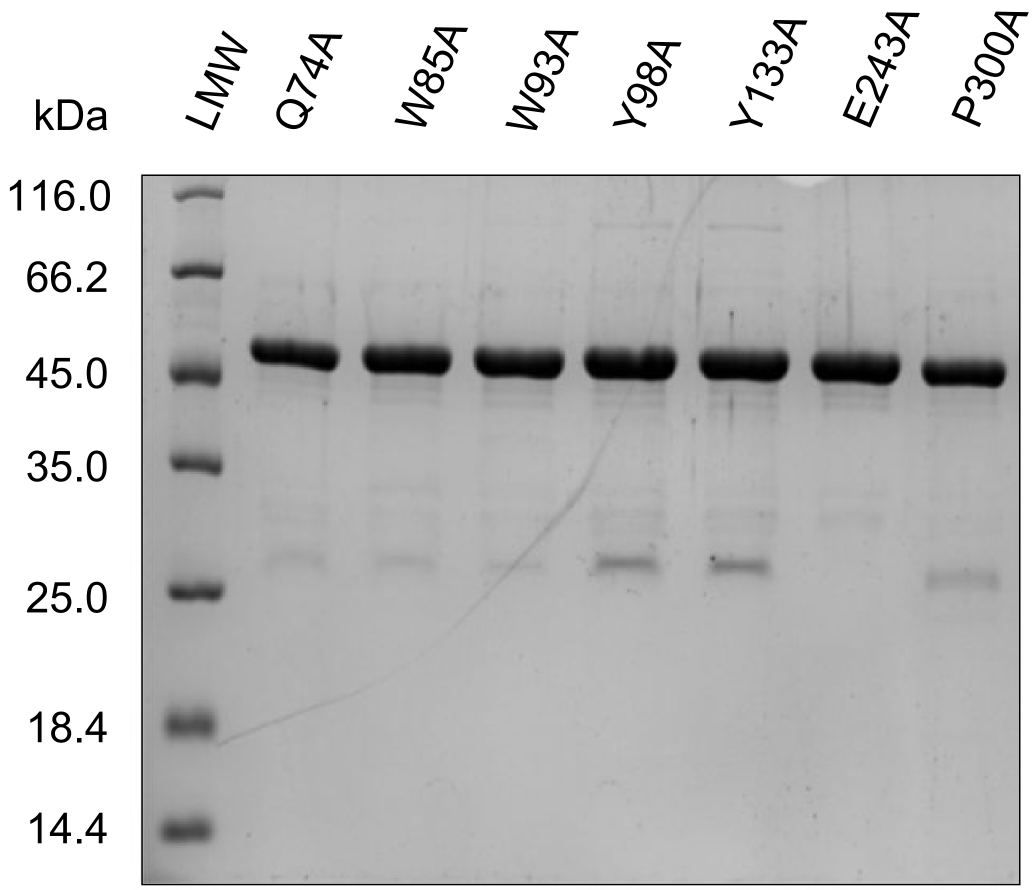

Figure S7: Assessment of the purity of *po*NdmH alanine mutants. After enrichment by IMAC and SEC, the purity of the proteins (3 µg each) was assessed via SDS-PAGE. LMW protein standard (Thermo Fisher Scientific) was used to estimate the molecular weight of the purified proteins, which are consistent with the theoretical values of ~51 kDa.

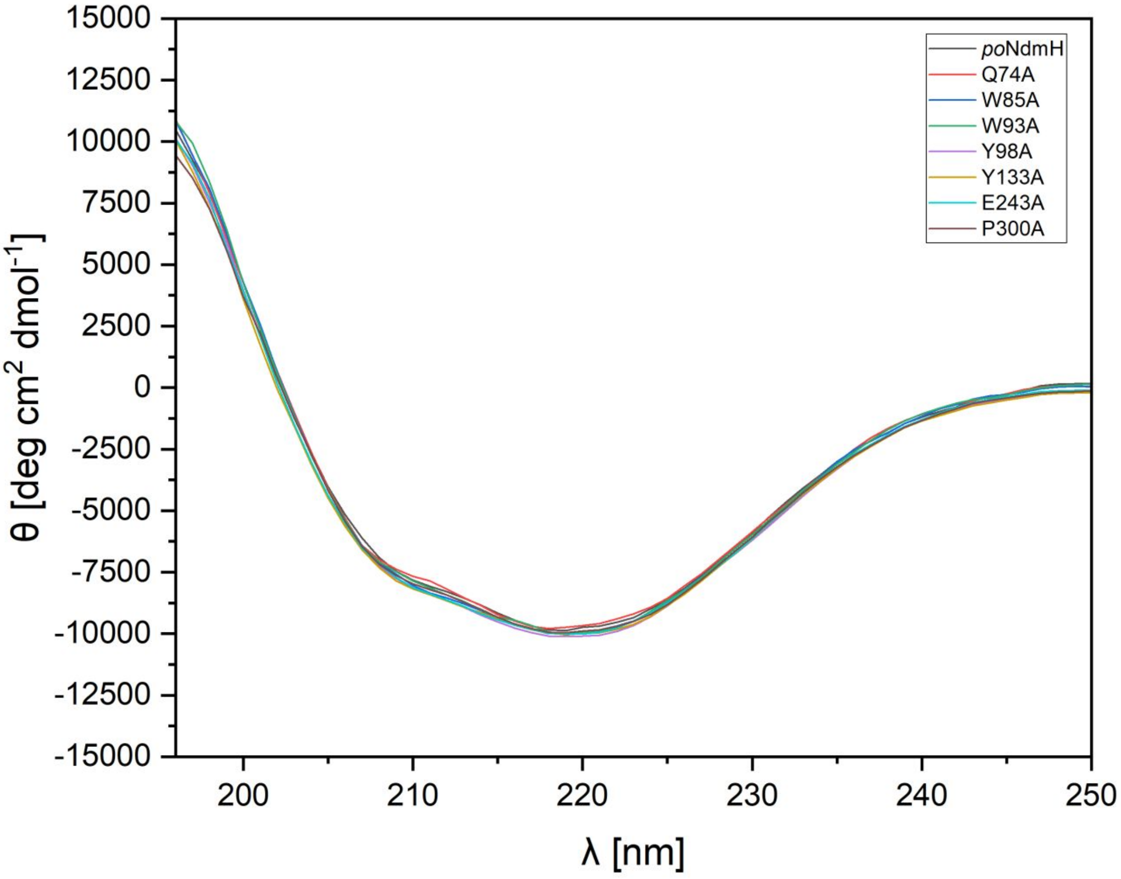

Figure S8: Assessment of secondary structure of purified *po*NdmH alanine mutants by far-UV circular dichroism (CD) spectroscopy. Far-UV CD spectra of 10 µM protein each were recorded in 50 mM Tris/HCl (pH 7.5), 50 mM KCl. Measurements were conducted in five replicas at 25 °C using a quartz cuvette (d = 0.2 mm). All variants show CD spectra that are characteristic of properly folded proteins and no significant differences between the spectra of the alanine mutants and wild-type *po*NdmH are observable.

*po*NdmH_W85A + N^2,N^2-Dimethylguanine

k_{cat}= 1.7 s^{-1}

K_M= 400 µM

k_{cat}/K_M= 4.3 * 10^3 $M^{-1}s^{-1}$

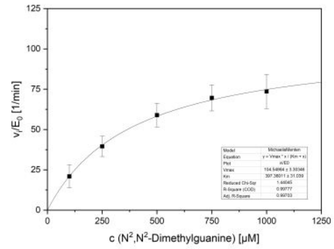

*po*NdmH_W93A + N^2,N^2-Dimethylguanine

k_{cat}= 3.9 s^{-1}

K_M= 550 µM

k_{cat}/K_M= 7.1 * 10^3 $M^{-1}s^{-1}$

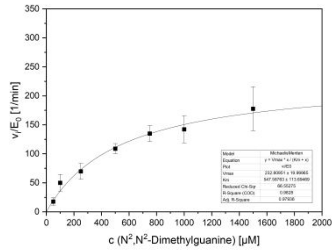

*po*NdmH_Y98A + N^2,N^2-Dimethylguanine

k_{cat}= 13.4 s^{-1}

K_M= 210 µM

k_{cat}/K_M= 6.4 * 10^4 $M^{-1}s^{-1}$

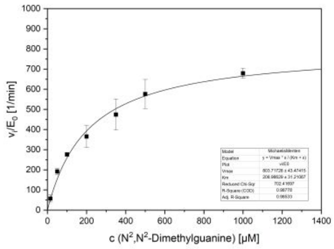

*po*NdmH_Y133A + N^2,N^2-Dimethylguanine

k_{cat}= 0.5 s^{-1}

K_M= 1930 µM

k_{cat}/K_M= 2.6 * 10^2 $M^{-1}s^{-1}$

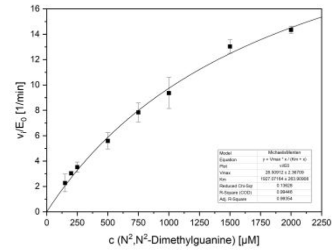

*po*NdmH_P300A + N^2,N^2-Dimethylguanine

k_{cat}= 6.3 s^{-1}

K_M= 205 µM

k_{cat}/K_M= 3.1 * 10^4 $M^{-1}s^{-1}$

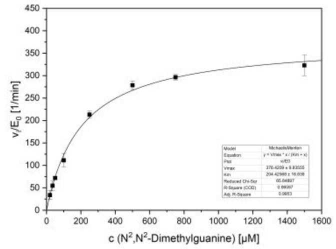

*po*NdmH_Q74A + N^2,N^2-Dimethylguanine

k_{cat}= n.d.

K_M= n.d.

k_{cat}/K_M= n.d.

*po*NdmH_E243A + N^2,N^2-Dimethylguanine

k_{cat}= n.d.

K_M= n.d.

k_{cat}/K_M= n.d.

Figure S9: Steady-state enzyme kinetics of *po*NdmH alanine mutants with N^2,N^2-dimethylguanine as substrate. The experimental conditions included 50 mM potassium phosphate (pH 7.5) and varying concentrations of N^2,N^2-dimethylguanine. The Michaelis constant K_M and the turnover number k_{cat} were obtained by fitting the data from triplicate measurements at 25 °C to the Michaelis-Menten equation using Origin 2022 (© OriginLab Corporation). Catalytic efficiencies (k_{cat}/K_M) of all enzymes are listed in Figure 4B. No activity was detectable for *po*NdmH_Q74A and *po*NdmH_E243A (n.d.).

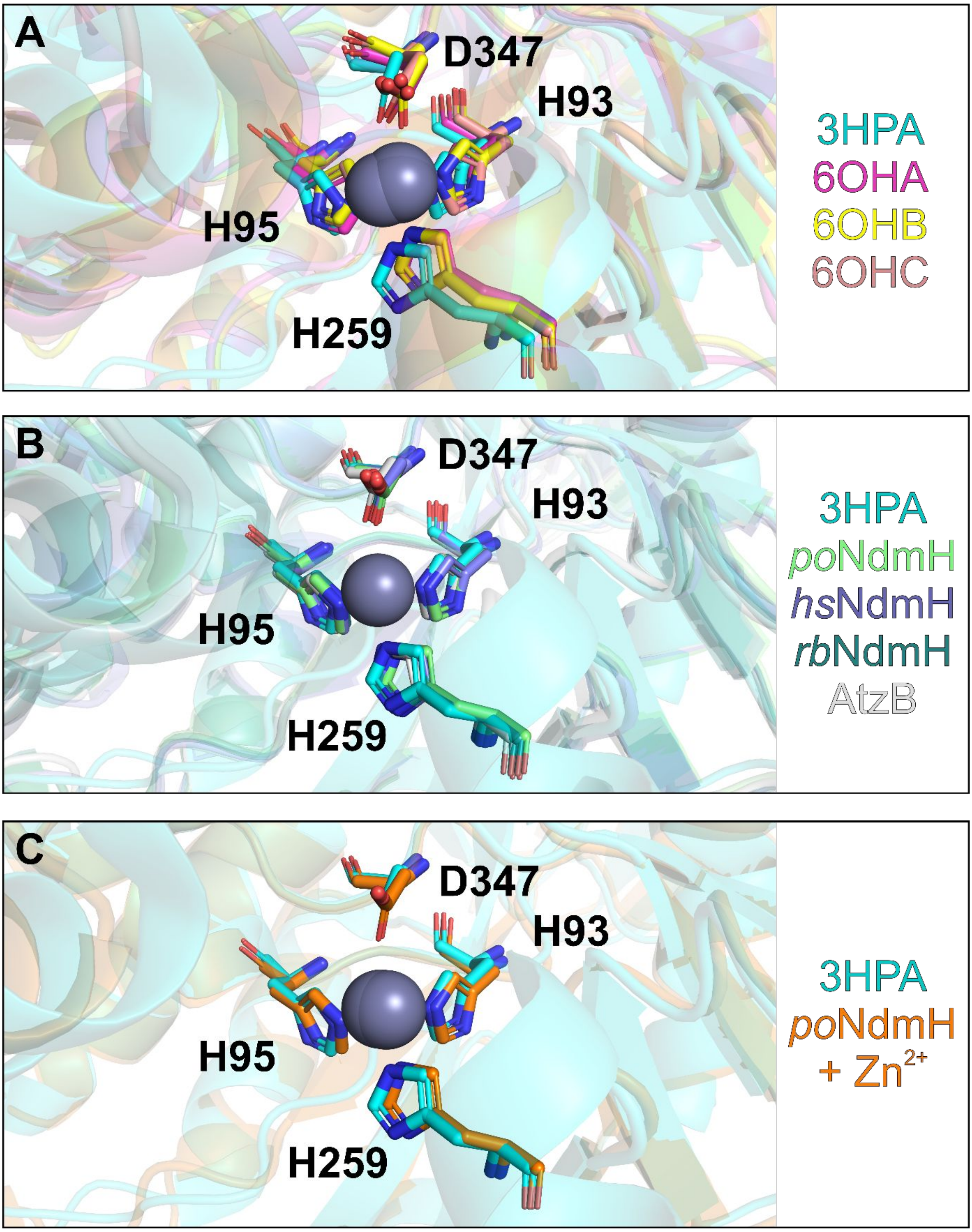

Figure S10: Metal-binding motif of AHS subtype III members. All enzymes investigated within this study belong to a recently analyzed AHS subtype III cluster of guanine deaminases.[1] While all AHS subtypes (except subtype VII) use divalent metal ions (either Zn^{2+}, Fe^{2+}, or Ni^{2+}) for catalysis, Zn^{2+} is the most prevalent metal ion mediating catalysis in AHS subtype III members.[6,7] (**A**) Several crystal structures of an AtzB-cluster member (PDB: 3HPA) and GuaD-cluster members (PDB: 6OHA, 6OHB, 6OHC) revealed the presence of divalent zinc in the active sites coordinated by three histidines and one asparatate, which constitute the metal-binding motif that is highly conserved among AHS subtype III enzymes.[6–8] The figure

shows a superposition of these PDB-available crystal structures. The zinc ions are depicted as spheres, while the coordinating residues are depicted as sticks. For clarity, only the metal-binding residues from 3HPA are labeled. (**B**) The same metal-binding residues are also present in NdmH and AtzB enzymes. The figure shows a superposition of AtzB-cluster members including an 8-OxoGuaD crystal structure (PDB: 3HPA) and AlphaFold3[9] apo models of the analyzed NdmH and AtzB enzymes. The zinc ion from 3HPA is depicted as sphere, while the coordinating residues are depicted as sticks. For clarity, only the metal-binding residues from 3HPA are labeled. Notably, for wild-type AtzB, which is 58-99% identical to the experimentally analyzed NdmH enzymes, a 1:1 zinc-to-subunit stoichiometry has been determined.[10] (**C**) The figure shows a superposition of 3HPA and an AlphaFold3[9] model of *po*NdmH with bound divalent zinc. The zinc ions are depicted as spheres, while the coordinating residues are depicted as sticks. For clarity, only the metal-binding residues from 3HPA are labeled. The shown D347 from 3HPA corresponds to D326 from *po*NdmH (cf. Figure 4C). Although the identity of the metal ion in NdmHs has not been determined experimentally, all these findings point to the presence of a divalent zinc ion within their active sites as characteristic for catalysis in AHS enzymes.

AtzB_I170N + N^2,N^2-Dimethylguanine

k_{cat} = 3.98 s^{-1}

K_M = 4251 µM

k_{cat}/K_M = 9.4 * 10^2 $M^{-1}s^{-1}$

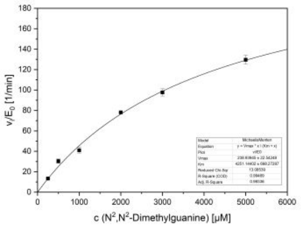

AtzB_S218C_S219Q + N^2,N^2-Dimethylguanine

k_{cat} = 9.32 s^{-1}

K_M = 925 µM

k_{cat}/K_M = 1.0 * 10^4 $M^{-1}s^{-1}$

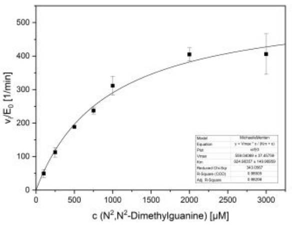

AtzB_S218C + N^2,N^2-Dimethylguanine

k_{cat} = 7.90 s^{-1}

K_M = 3651 µM

k_{cat}/K_M = 2.2 * 10^3 $M^{-1}s^{-1}$

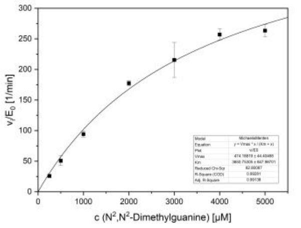

AtzB_S218C_I222N + N^2,N^2-Dimethylguanine

k_{cat} = 1.37 s^{-1}

K_M = 4487 µM

k_{cat}/K_M = 3.0 * 10^2 $M^{-1}s^{-1}$

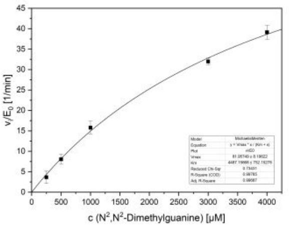

AtzB_S219Q + N^2,N^2-Dimethylguanine

k_{cat} = 3.47 s^{-1}

K_M = 2917 µM

k_{cat}/K_M = 1.2 * 10^3 $M^{-1}s^{-1}$

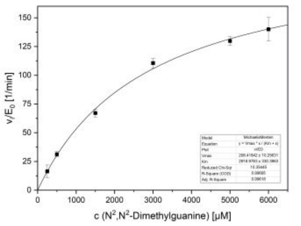

AtzB_S219Q_I222N + N^2,N^2-Dimethylguanine

k_{cat} = 0.82 s^{-1}

K_M = 279 µM

k_{cat}/K_M = 2.9 * 10^3 $M^{-1}s^{-1}$

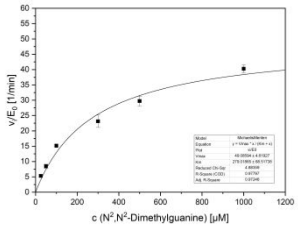

AtzB_I222N + N^2,N^2-Dimethylguanine

k_{cat} = 0.99 s^{-1}

K_M = 4668 µM

k_{cat}/K_M = 2.1 * 10^2 $M^{-1}s^{-1}$

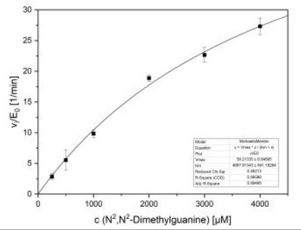

AtzB_I170N_S218C_S219Q + N^2,N^2-Dimethylguanine

k_{cat} = 5.40 s^{-1}

K_M = 476 µM

k_{cat}/K_M = 1.1 * 10^4 $M^{-1}s^{-1}$

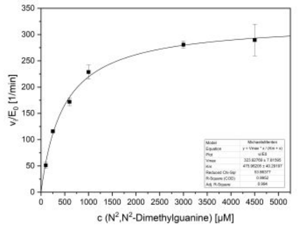

AtzB_I170N_S218C + N^2,N^2-Dimethylguanine

k_{cat} = 7.48 s^{-1}

K_M = 2927 µM

k_{cat}/K_M = 2.6 * 10^3 $M^{-1}s^{-1}$

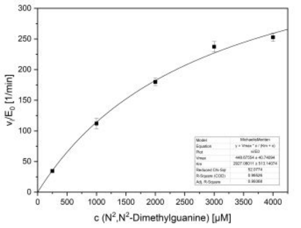

AtzB_I170N_S218C_I222N + N^2,N^2-Dimethylguanine

k_{cat} = 17.35 s^{-1}

K_M = 732 µM

k_{cat}/K_M = 2.4 * 10^4 $M^{-1}s^{-1}$

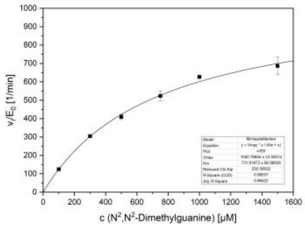

AtzB_I170N_S219Q + N^2,N^2-Dimethylguanine

k_{cat} = 1.52 s^{-1}

K_M = 610 µM

k_{cat}/K_M = 2.5 * 10^3 $M^{-1}s^{-1}$

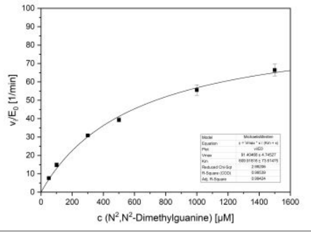

AtzB_I170N_S219Q_I222N + N^2,N^2-Dimethylguanine

k_{cat} = 7.12 s^{-1}

K_M = 675 µM

k_{cat}/K_M = 1.1 * 10^4 $M^{-1}s^{-1}$

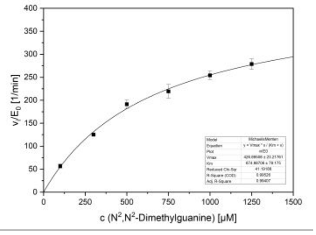

AtzB_I170N_I222N + N^2,N^2-Dimethylguanine

k_{cat} = 2.04 s^{-1}

K_M = 1189 µM

k_{cat}/K_M = 1.7 * 10^3 $M^{-1}s^{-1}$

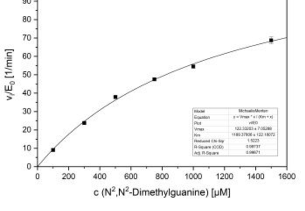

AtzB_S218C_S219Q_I222N + N^2,N^2-Dimethylguanine

k_{cat} = 3.98 s^{-1}

K_M = 2219 µM

k_{cat}/K_M = 1.8 * 10^3 $M^{-1}s^{-1}$

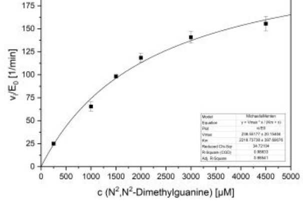

Figure S11: Steady-state enzyme kinetics of previously[3] generated AtzB mutants with N^2,N^2-dimethylguanine as substrate. The experimental conditions included 50 mM potassium phosphate (pH 7.5) and varying concentrations of N^2,N^2-dimethylguanine. The Michaelis constant K_M and the turnover number k_{cat} were obtained by fitting the data from triplicate measurements at 25 °C to the Michaelis-Menten equation using Origin 2022 (© OriginLab Corporation). Values for guanine and hydroxyatrazine hydrolysis were determined in our previous study (cf. Figure 5A, 5B).[3]

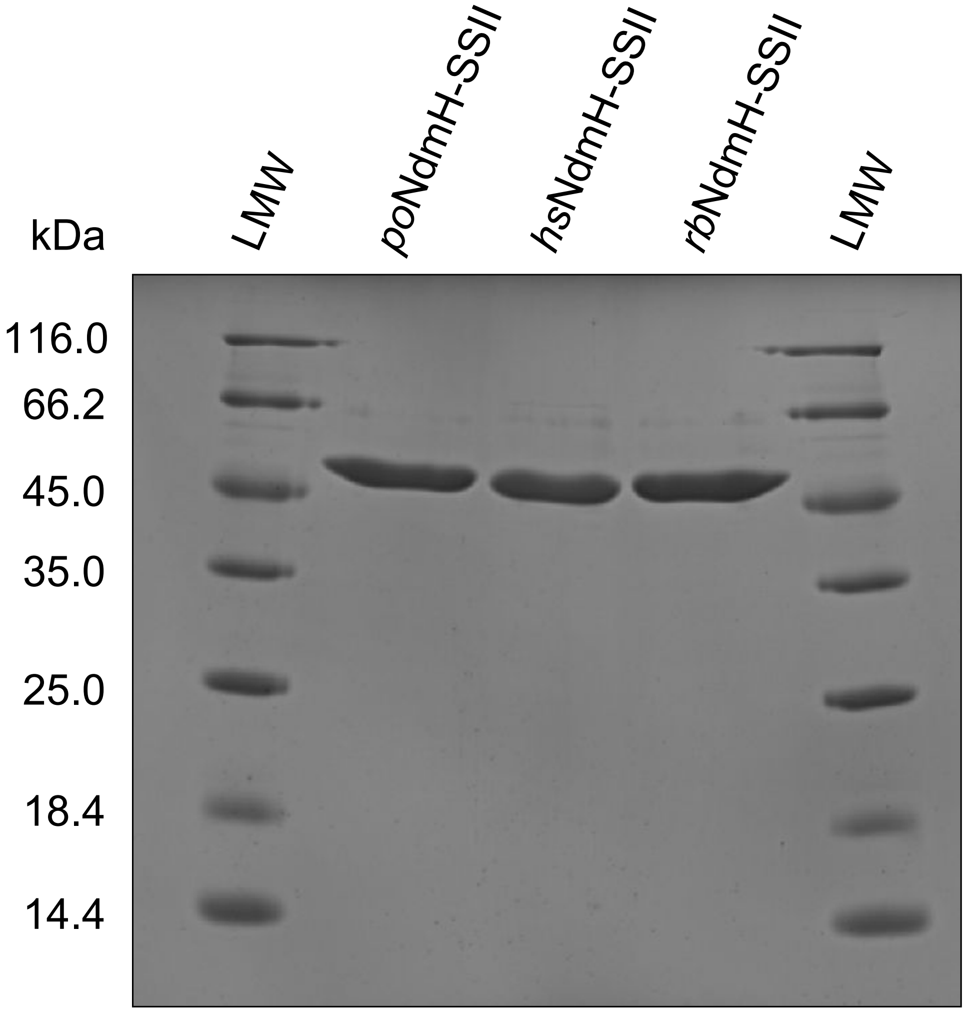

Figure S12: Assessment of the purity of NdmH-SSII mutants. After enrichment by IMAC and SEC, the purity of the proteins (3 µg each) was assessed via SDS-PAGE. LMW protein standard (Thermo Fisher Scientific) was used to estimate the molecular weight of the purified proteins, which are consistent with the theoretical values of ~50 kDa.

*po*NdmH-SSII + N^2,N^2-Dimethylguanine

k_{cat} = 7.04 s^{-1}

K_M = 1023 µM

k_{cat}/K_M = 6.8 * 10^3 $M^{-1}s^{-1}$

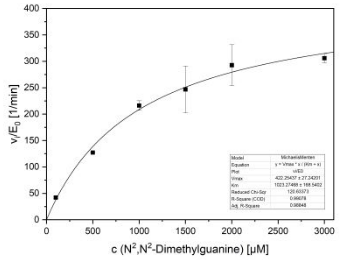

*po*NdmH-SSII + Hydroxyatrazine

k_{cat} = 7.53 s^{-1}

K_M = 28.4 µM

k_{cat}/K_M = 2.7 * 10^5 $M^{-1}s^{-1}$

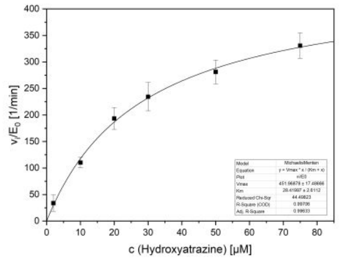

*hs*NdmH-SSII+ N^2,N^2-Dimethylguanine

k_{cat} = 0.5 s^{-1}

K_M = 1848 µM

k_{cat}/K_M = 2.7 * 10^2 $M^{-1}s^{-1}$

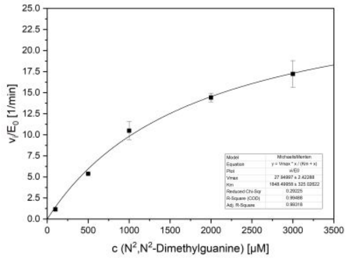

*hs*NdmH-SSII+ Hydroxyatrazine

k_{cat} = 0.44 s^{-1}

K_M = 19.1 µM

k_{cat}/K_M = 2.3 * 10^4 $M^{-1}s^{-1}$

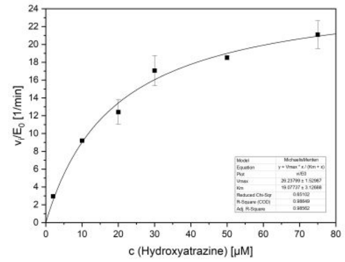

*rb*NdmH + Hydroxyatrazine

k_{cat} = n.d.

K_M = n.d.

k_{cat}/K_M = 4.1 * 10^1 $M^{-1}s^{-1}$

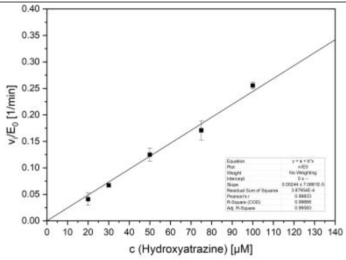

*rb*NdmH-SSII + Hydroxyatrazine

k_{cat} = 5.9 s^{-1}

K_M = 15.8 µM

k_{cat}/K_M = 3.7 * 10^5 $M^{-1}s^{-1}$

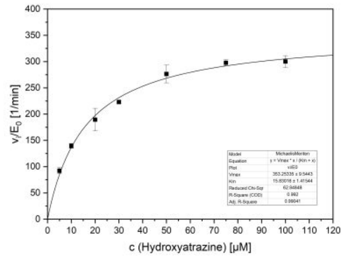

*rb*NdmH-SSII + N^2,N^2-Dimethylguanine

k_{cat} = 2.9 s^{-1}

K_M = 823 µM

k_{cat}/K_M = 3.5 * 10^3 $M^{-1}s^{-1}$

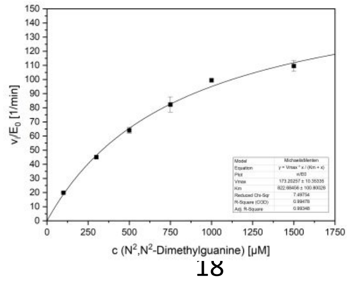

Figure S13: Steady-state enzyme kinetics of several NdmH variants with N^2,N^2-dimethylguanine and hydroxyatrazine as substrates. The experimental conditions included 50 mM potassium phosphate (pH 7.5) and varying concentrations of N^2,N^2-dimethylguanine or hydroxyatrazine, respectively. The Michaelis constant K_M and the turnover number k_{cat} were obtained by fitting the data from triplicate measurements at 25 °C to the Michaelis-Menten equation using Origin 2022 (© OriginLab Corporation). For *rb*NdmH, k_{cat}/K_M for hydroxyatrazine was calculated from the slope of the curve after fitting the data to a linear equation. Values for hydroxyatrazine hydrolysis by *po*NdmH, *hs*NdmH, AtzB, and AtzB-CQNN were determined in our previous study (cf. Figure 5C).[3]

Arrhenius plot

$\ln(k) = \ln(A) - E_a/R * 1/T$

AtzB-CQNN + Hydroxyatrazine

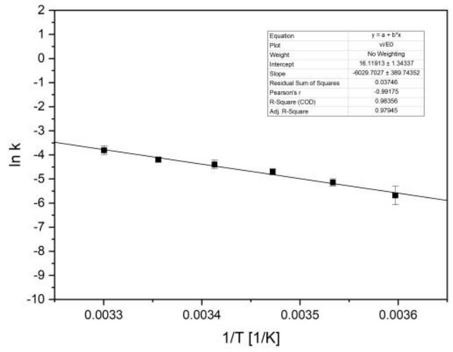

AtzB-CQNN + N^2,N^2-Dimethylguanine

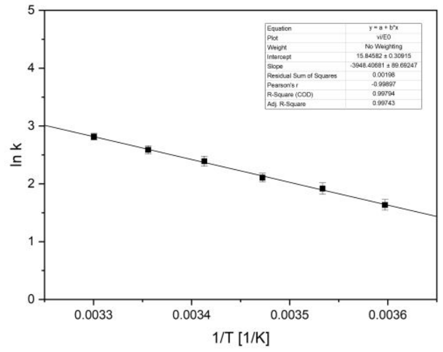

AtzB + Hydroxyatrazine

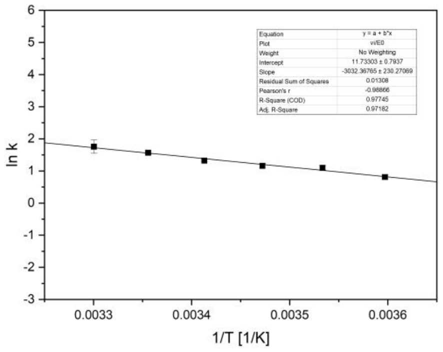

AtzB + N^2,N^2-Dimethylguanine

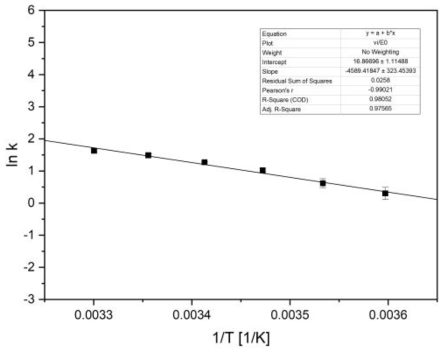

Eyring plot

$\ln(k/T) = \ln(\kappa * k_B/h) + \Delta S^{‡}/R - \Delta H^{‡}/R * 1/T$

AtzB-CQNN + Hydroxyatrazine

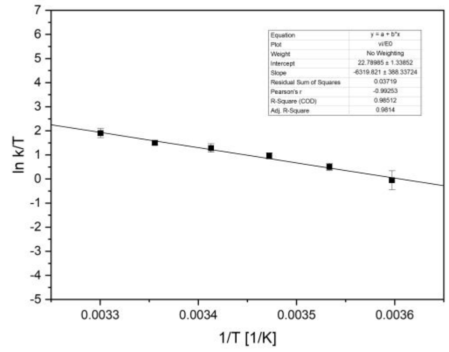

AtzB-CQNN + N^2,N^2-Dimethylguanine

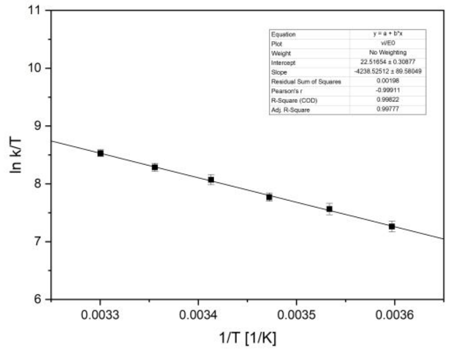

AtzB + Hydroxyatrazine

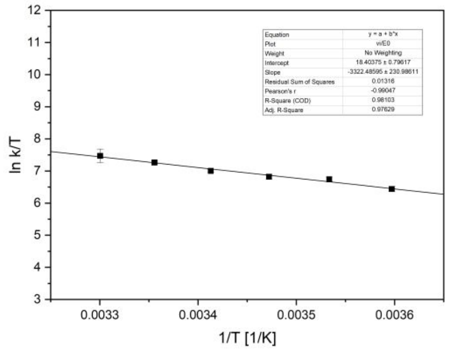

AtzB + N^2,N^2-Dimethylguanine

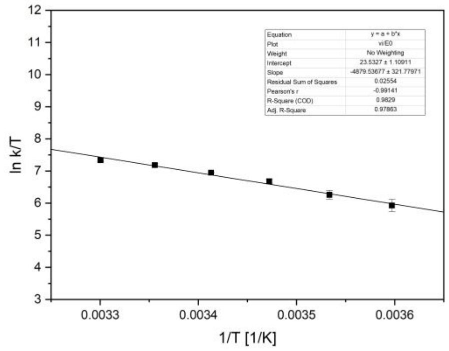

Figure S14: Temperature-dependent activity measurements (278 K - 303 K) for the hydrolysis of hydroxyatrazine and N^2,N^2-dimethylguanine by wild-type AtzB and AtzB-CQNN. Triplicate measurements were performed at 278 K, 283 K, 288 K, 293 K, 298 K, and 303 K. The experimental conditions included 50 mM potassium phosphate (pH 7.5) and 2 mM N^2,N^2-dimethylguanine or 0.1 mM hydroxyatrazine, respectively. For each temperature, k_{cat} was determined using the Michaelis-Menten equation. Then, values for the activation energy (E_a), the enthalpy of activation ($\Delta H^{ǂ}$), the entropy of activation ($\Delta S^{ǂ}$), and the Gibbs energy of activation ($\Delta G^{ǂ\,(298)}$) were obtained (cf. Figure 5D) by fitting the data to the linear Arrhenius and Eyring equation (framed with boxes) using Origin 2022 (© OriginLab Corporation). E_a was calculated from the slope of the Arrhenius plot, while $\Delta H^{ǂ}$ was calculated from the slope of the Eyring plot. $\Delta S^{ǂ}$ was calculated from the y-intercept of the Eyring plot using a value of 0.8 for κ.[11–13] Then, $T^*\Delta S^{ǂ\,(298)}$ was calculated as 298 $K^*\Delta S^{ǂ}$, while $\Delta G^{ǂ\,(298)}$ was calculated as $\Delta H^{ǂ}$-298 $K^*\Delta S^{ǂ\,(298)}$. All determined values are listed in Figure 5D. These analyses yielded similar values for E_a and $\Delta H^{ǂ}$ as inferred from the Arrhenius and Eyring plots, respectively. Moreover, for each enzyme-substrate pair the enthalpic contribution ($\Delta H^{ǂ}$) to $\Delta G^{ǂ\,(298)}$ exceeds the entropic contribution ($T^*\Delta S^{ǂ\,(298)}$). Interestingly, for N^2,N^2-dimethylguanine the difference in enthalpy of activation ($\Delta\Delta H^{ǂ}$ = +5.4 kJ/mol), the difference in entropy of activation ($T^*\Delta\Delta S^{ǂ\,(298)}$ = +2.5 kJ/mol), and the difference in Gibbs energy of activation ($\Delta\Delta G^{ǂ\,(298)}$ = +2.9 kJ/mol) for the evolutionary process (AtzB-CQNN → AtzB) point to both a moderate enthalpic deterioration and entropic improvement resulting in subtly diminished rate constants. By contrast, for hydroxyatrazine the corresponding values for $\Delta\Delta H^{ǂ}$ (-24.9 kJ/mol), $T^*\Delta\Delta S^{ǂ\,(298)}$ (-10.85 kJ/mol), and $\Delta\Delta G^{ǂ\,(298)}$ (-14.1 kJ/mol) for the evolutionary process (AtzB-CQNN → AtzB) point to a strong enthalpic improvement accompanied by a moderate entropic deterioration of the catalyst.

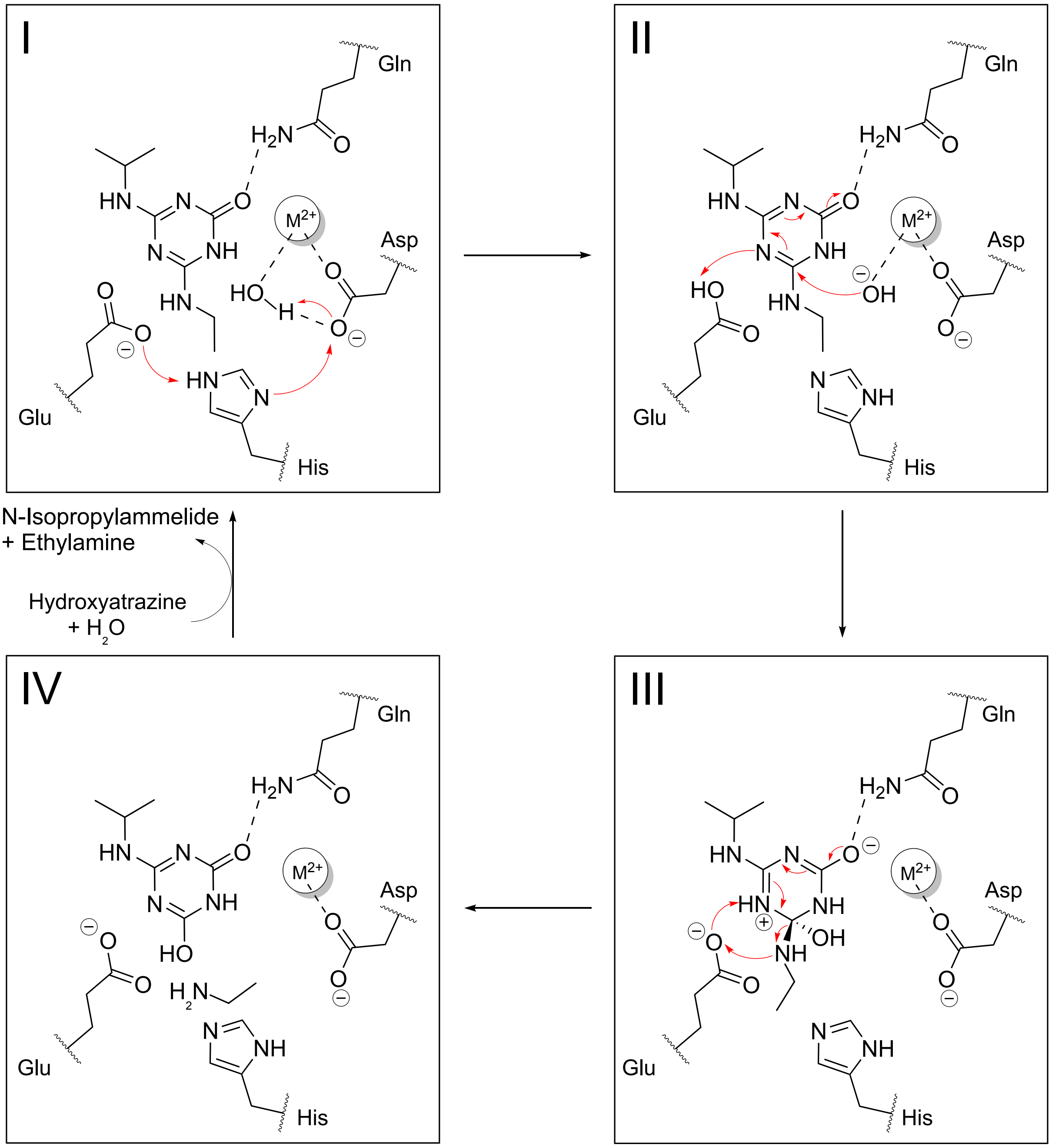

Figure S15: The proposed reaction mechanism for the hydrolysis of hydroxyatrazine by AtzB and homologues[1,3] can be divided into four steps: **I**: The water nucleophile is deprotonated by an aspartate and the proton is shuttled from the aspartate to a glutamate by a bridging histidine. **II**: Hydroxyatrazine is protonated by the glutamate and the nucleophile attacks the C=N double bond of hydroxyatrazine at the Bürgi-Dunitz angle (107°) through a 1,6 conjugate addition. **III**: A new stereocenter is formed within the tetrahedral intermediate. Shown is the zwitterionic resonance structure of the intermediate in which the oxyanion is stabilized by a glutamine. The glutamate deprotonates the ring nitrogen and protonates the ethylamine substituent resulting in a backflow of π-electrons. **IV**: The leaving group is released and the product N-isopropylammelide is formed. Electron flows and divalent metal ions are represented by red arrows and M^{2+}, respectively.

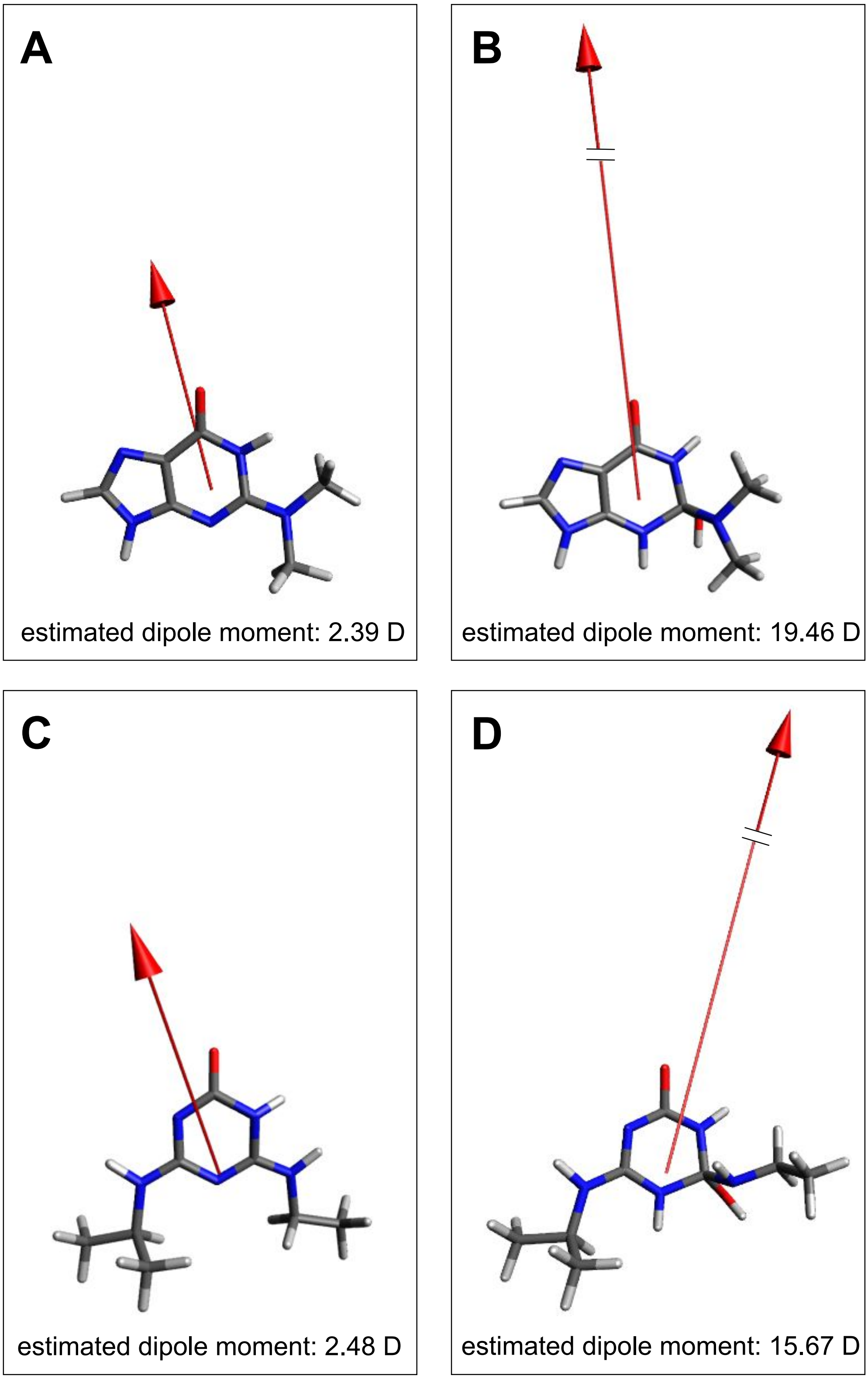

Figure S16: Estimation of dipole moments. The dipole moments of (**A**) the free substrate N^2,N^2-dimethylguanine (cf. Figure 4C-I), (**B**) the free tetrahedral intermediate of N^2,N^2-dimethylguanine hydrolysis (cf. Figure 4C-III), (**C**) the free substrate hydroxyatrazine (cf. Figure S15-I), and (**D**) the free tetrahedral intermediate of hydroxyatrazine hydrolysis (cf. Figure S15-III) were estimated and visualized via Avogadro[14] after energy-minimization of the bare molecules individually by employing the MM2 force field in Chem3D using standard parameters.

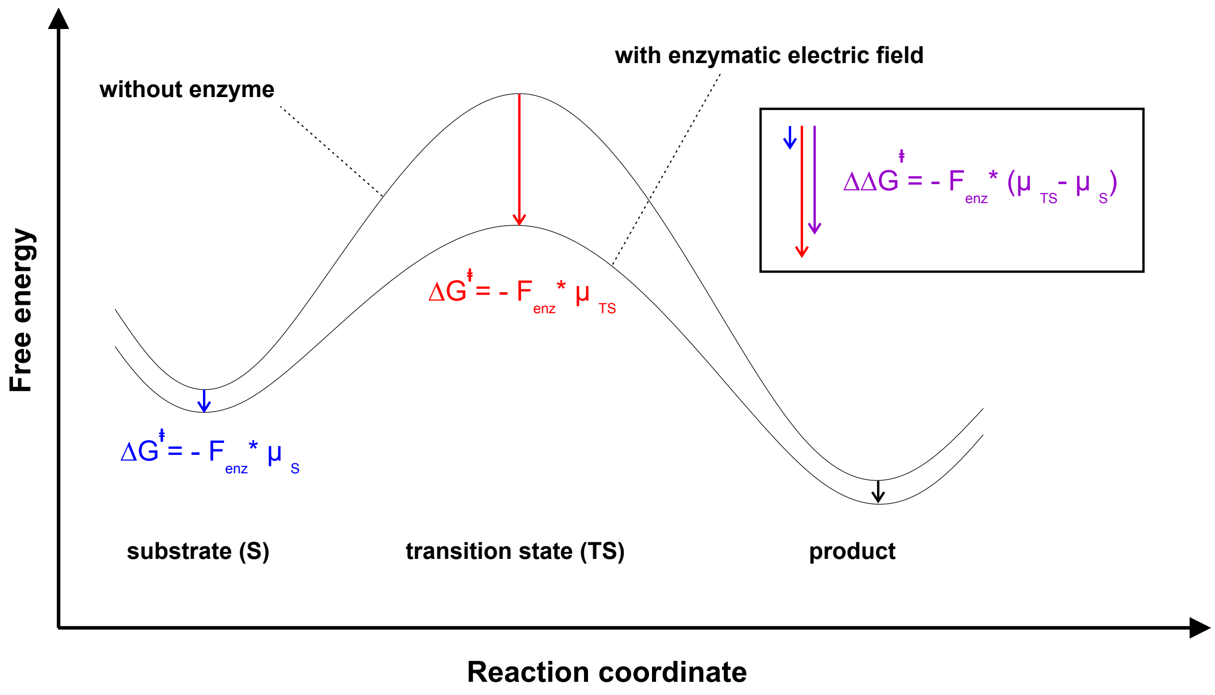

Figure S17: Reaction scheme for electric field catalysis. Electric field catalysis is defined by an electric field environment which stabilizes the dipole moment of the transition state μ_{TS} more than the dipole moment of the substrate μ_S. Here, $\Delta\Delta G^{‡}$ refers to the reduction in free energy barrier and is caused by F_{enz}, which is the force exerted by the enzymatic electric field.[11,15–17]

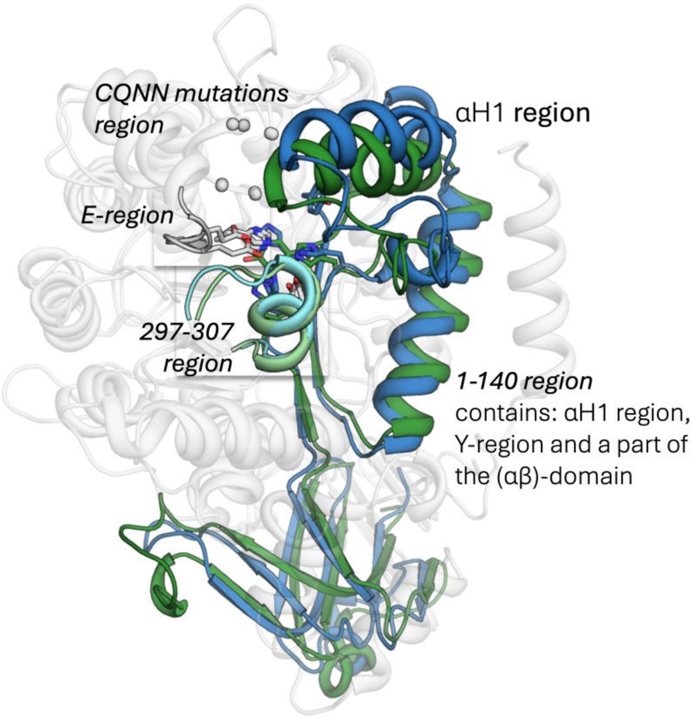

Figure S18: **Structural superposition of *po*NdmH and *po*GuaD.** Overlay of *po*NdmH and *po*GuaD structures with the regions exhibiting elevated flexibility in the RMSF analysis (cf. Figure 6) highlighted. Residues 1-140 are shown in dark green (*po*NdmH) and dark blue (*po*GuaD), while residues 297-307 are shown in light green (*po*NdmH) and cyan (*po*GuaD). The 1-140 segment contains part of the (αβ)-domain, the Y-region, and the αH1 helix. Catalytic residues are shown as sticks for clarity. The E-region and the CQNN mutations region are also highlighted.

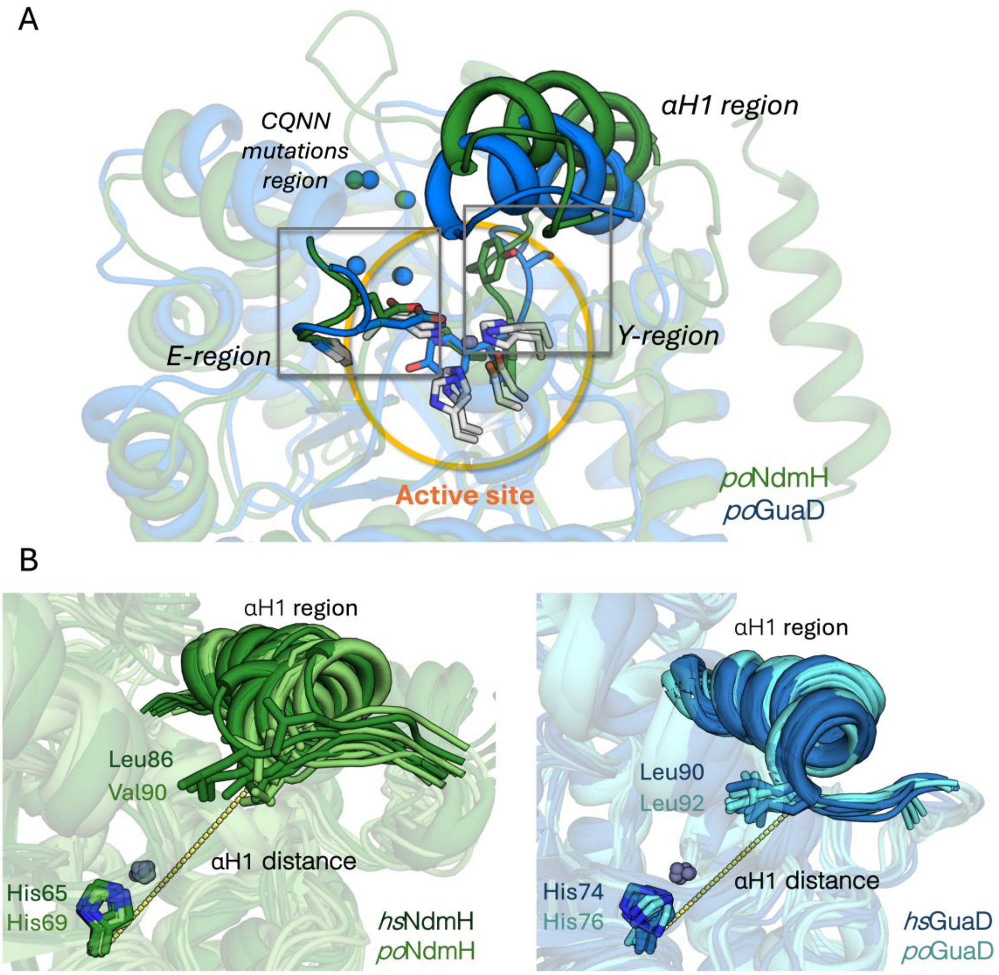

Figure S19: Key structural regions of the conformational landscapes analysis (cf. Figure 7). (**A**) Key E- and Y-regions defining the active site principal component (PC, y axis) are highlighted using gray boxes (*po*NdmH in green, and *po*GuaD in blue). The key α helix 1 (αH1), which defines the x axis of the conformational landscapes in Figure 7 is also indicated. All contributing regions are located near the active site (orange circle). The CQNN mutations region is additionally shown. (**B**) Overlay of representative structures extracted from the conformational landscapes. NdmH systems (left panel, *hs*NdmH and *po*NdmH in dark green and light green, respectively) show a larger αH1 conformational heterogeneity than GuaD systems (right panel, *hs*GuaD and *po*GuaD in dark blue and light blue, respectively). The αH1 distance is defined between Cα-His and Cα-Leu/Val in αH1, both shown as sticks.

MD simulation details:

MD equilibration phase was done following the protocol described by Roe and Brooks with small differences fine-tuned to our systems.[18] The bonds involving hydrogen are constrained by the SHAKE algorithm during the non-minimization steps. Long-range electrostatic effects were modeled using the particle mesh-Ewald method.[19] For Lennard–Jones and electrostatic interactions, a 10 Å cut-off was applied. The MD protocol starts with the minimization phase of 1500 steps of the steepest descent method followed by 3500 steps of the conjugate gradient method with a positional restrain (*i.e.*, a force constant of 5.0 $kcal \cdot mol^{-1} \cdot Å^{-2}$) to the protein heavy atoms. In the following heating phase a temperature increment from 25 K to 300K during 20 ps of MD simulation time, a Langevin thermostat with a collision frequency of 5 ps^{-1}, and a positional restrain (*i.e.*, a force constant of 5.0 $kcal \cdot mol^{-1} \cdot Å^{-2}$) to the protein heavy atoms are performed. A minimization and heating of all atoms in the system is the following step. This starts with two minimization stages of 1000 steps of the steepest descent method followed by 1500 steps of the conjugate gradient method each with a positional restrain (*i.e.*, force constant of 2.0 $kcal \cdot mol^{-1} \cdot Å^{-2}$ in the first minimization and 0.1 $kcal \cdot mol^{-1} \cdot Å^{-2}$ in the second) to the protein heavy atoms. Following, a third minimization phase of 1500 steps of the steepest descent method followed by 3500 steps of the conjugate gradient method without any positional restraint is performed. The system is then heated in accordance with the previously established procedure. Finally, a five-round equilibration phase at the NPT ensemble with a constant pressure of 1 atm is performed. The first four rounds were done with the Berendsen barostat, whereas the fifth one was done with a Monte-Carlo barostat. For all equilibration rounds, Langevin thermostat with a collision frequency of 1 ps^{-1} was used. A positional restraint to the protein-heavy atoms with a force constant of 1.0 and 0.5 $kcal \cdot mol^{-1} \cdot Å^{-2}$ was applied to the first and second equilibration rounds, respectively. In the third round of 10 ps equilibration, a positional restraint to the backbone-heavy atoms with a force constant of 0.5 $kcal \cdot mol^{-1} \cdot Å^{-2}$ was used. The fourth and fifth equilibration of 10 ps and 1 ns, respectively, were performed without any restraint. The production runs were performed at the NVT ensemble with the Langevin thermostat with a collision frequency of 1 ps^{-1} during 100 ns for all systems. A total of 5 replicas of equilibration and production runs were performed reaching a total simulation time of 0.5 µs/system (5 replicas x 100 ns) for *hs*NdmH, *po*NdmH, *hs*GuaD, and *po*GuaD. The MD trajectories were analyzed using the Python packages MDTraj,[20] pytraj which is part of the cpptraj package,[18] MDAnalysis, and PyEMMA.[21]

Table S1: Catalytic efficiencies k_{cat}/K_M determined under steady-state kinetic conditions.

k_{cat}/K_M [M^{-1} s^{-1}]	AtzB	*po*NdmH	*hs*NdmH	*rb*NdmH	*pa*8-Oxo-GuaD	*ec*GuaD	*po*GuaD	*hs*GuaD
N^2,N^2-Dimethyl-guanine **1**	820[a]	540000[a]	170000[a]	940000[a]	-	-	-	-
Guanine **2**	n.d.[b] /	2200[b]	1100[b]	260[a]	1300[c]	210000[c]	2400000[a]	1600000[a]
Hydroxy-atrazine **3**	240000[b]	42[b] /	n.d.[b] /	41[a] /	-	-	-	-
Ammeline **4**	/		/		n.d.[c]	830[c]		
8-Oxoguanine **5**	-		-		20000[c]	-	-	-
Isocytosine **9**	/	/	-	/	2200[c]	-	/	/

[a]: determined within the present study
[b]: determined in our previous study[3]
[c]: determined elsewhere[4,5,8,22]

n.d.: no activity detectable under steady-state kinetic conditions
-: no activity detectable in HPLC endpoint assays (Figure 2B)
/: partial turnover detectable in HPLC endpoint assays (Figure 2B)

For a large subset of substrates hydrolyzed by enzymes analyzed in the present study (cf. Figure 2B), kinetic measurements under steady-state conditions were conducted. Activities for enzyme-substrate combinations in which only partial substrate turnover was observed via HPLC after a reaction period of 24 h (Figure 2B, yellow squares) can be considered as promiscuous: This conclusion is supported by the presented steady-state kinetic measurements of *e.g.* **2** + AtzB, **3** + *po*NdmH, **3** + *hs*NdmH, and **3** + *rb*NdmH. Moreover, steady-state kinetics for *pa*8-OxoGuaD and *ec*GuaD have been reported in previous studies, establishing **5** and **2** as their primary substrates, respectively, while our previous and present study corroborate **3** and **1** as the primary substrates of AtzB and NdmHs, respectively.

Table S2: Characteristics and amino acid sequences of the analyzed AHS enzymes.

Protein	MW [Da]	ε_{280} [$M^{-1}cm^{-1}$]	Number of amino acids	Sequence
***ec*GuaD (from *<u>E</u>scherichia <u>c</u>oli*)**	51309	76780	447	MMSGEHTLKAVRGSFIDVTRTIDNPEEIASA<br>LRFIEDGLLLIKQGKVEWFGEWENGKHQI<br>PDTIRVRDYRGKLIVPGFVDTHIHYPQSEMV<br>GAYGEQLLEWLNKHTFPTERRYEDLEYAR<br>EMSAFFIKQLLRNGTTTALVFGTVHPQSVD<br>ALFEAASHINMRMIAGKVMMDRNAPDYLLD<br>TAESSYHQSKELIERWHKNGRLLYAITPRFA<br>PTSSPEQMAMAQRLKEEYPDTWVHTHLCE<br>NKDEIAWVKSLYPDHDGYLDVYHQYGLTGK<br>NCVFAHCVHLEEKEWDRLSETKSSIAFCPT<br>SNLYLGSGLFNLKKAWQKKVKVGMGTDIGA<br>GTTFNMLQTLNEAYKVLQLQGYRLSAYEAF<br>YLATLGGAKSLGLDDLIGNFLPGKEADFVV<br>MEPTATPLQQLRYDNSVSLVDKLFVMMTLG<br>DDRSIYRTYVDGRLVYERNLEHHHHHH
***pa*8-OxoGuaD (from *<u>P</u>seudomonas <u>a</u>eruginosa*)**	49368	54430	457	MSRTWIRNPLAIFTANGLDAAGGLVVEDGRI<br>VELLGAGQQPAQPCASQFDASRHVVLPGL<br>VNTHHHFYQTLTRAWAPVVNQPLFPWLKTL<br>YPVWARLTPEKLELATKVALAELLLSGCTT<br>AADHHYLFPGGLEQAIDVQAGVVEELGMRA<br>MLTRGSMSLGEKDGGLPPQQTVQEAETILA<br>DSERLIARYHQRGDGARVQIALAPCSPFSV<br>TPEIMRASAEVAARHDVRLHTHLAETLDEE<br>DFCLQRFGLRTVDYLDSVGWLGPRTWLAH<br>GIHFNAEEIRRLGEAGTGICHCPSSNMRLAS<br>GICPTVELEAAGAPIGLGVDGSASNDASNMI<br>LEARQALYLQRLRYGAERITPELALGWAT<br>RGSARLLGRSDIGELAPGKQADLALFKLDEL<br>RFSGSHDPLSALLLCAADRADRVMVGGAW<br>RVVDGAVEGLDLAALIARHRAAASALIAGLE<br>HHHHHH
***hs*GuaD (previously[1] 'WP_135441580') (from *<u>H</u>aliea <u>sp</u>.* SAOS-164)**	47464	46870	438	MHHHHHHLDMGSGILRGRILHCIDTPGADG<br>AGVEYLEDGVLQFEDGVITLLADAREASAG<br>GLQLGDVPHLGAGLIVPGFIDTHVHAPQLAI<br>LGSYGEQLMAWLERYTFPAEARFADPDYA<br>AAAMDEFLGEMLRHGTTSALVFSTSHEDAT<br>EALFTAARARDLRLVAGKVLMDRNAPQGLL<br>DTAASGEAASRRLIERWHGAGRLAYAVTPR<br>FSITCSDEQLAAAGRLLRDYPGVYLQTHLAE<br>NPGEIAAVAELFPDAAHYLDTYDRHGLCGE<br>RSFFAHCVHLQPDELERLAATDSRVSLCPS<br>SNMFLGSGLYDWAQLEREGVCISLGSDVG<br>AGTSLSMLRTLGDAYRVCQLQEMSLPPMQ<br>GLYAVTLGNARALGVAERIGNLAVGSEADF<br>LVLDPAGNPQVQRRLQDVSDIDEEWFVYM<br>MLGDERLVASTWVAGREVVR

***po*GuaD (previously[1] 'WP_026789444') (from *Pleomorphomonas oryzae*)**	49753	31860	455	MHHHHHHLDMTFSDRFPSDRTLVRGRVLS FKRRPQRAGDTDAYTYLEDGVIVIDAGKVT DVIDASEIGRVGGKGVVLHDFSGKLILPGFID THIHFPQTQVIASYGEQLLEWLTRYTFPAES RYGDPAFAAAQARFFIDELLRNGTTTAVCY GSVHKGAAEALLTESERRGTAMFVGKTAM DRNAPPDVLDTAQSAYDDTASLITAWHGRG RQKVVITPRFAITSTPEQLEALGNLARAHPD CLVQTHLSENLEEIATVERLFPERSDYLDVY DHYGLVGPKSLMGHAIHLTPREIVRMSESG AVAVFCPTSNLFIGSGLFDYKGLEGEPYPV RIALATDVGGGTSYSMLATAAEAYKVMQLR GQKLSAIEAFHLMTRGNAEALGEPDLGRIEP GAHADLVVLDSTARPAMAHRLAAGNCDLE EELFVLMTLGGEQNVQEVFIGGQPQGLHRA DE
**AtzB (from *Pseudomonas sp.* strain ADP)**	53179	49390	489	MTTTLYTGFHQLVTGDVAGTVLNGVDILVR DGEIIGLGPDLPRTLAPIGVGQEQGVEVVN CRGLTAYPGLINTHHHFFQAFVRNLAPLDW TQLDVLAWLRKIYPVFALVDEDCIYHSTVV SMAELIKHGCTTAFDHQYNYSRRGGPFLVD RQFDAANLLGLRFHAGRGCITLPMAEGSTI PDAMRESTDTFLADCERLVSRFHDPRPFA MQRVVVAPSSPVIAYPETFVESARLARHLG VSLHTHLGEGETPAMVARFGERSLDWCEN RGFVGPDVWLAHGWEFTAADIARLAATGT GVAHCPAPVFLVGAEVTDIPAMAAAGVRVG FGVDGHASNDSSNLAECIRLAYLLQCLKAS ERQHPVPAPYDFLRMATQGGADCLNRPDL GALAVGRAADFFAVDLNRIEYIGANHDPRSL PAKVGFSGPVDMTVINGKVVWRNGEFPGL DEMELARAADGVFRRVIYGDPLVAALRRGT GVTPCLEHHHHHH
***hs*NdmH (previously[1,3] 'AtzB_Hom_Hal') (from *Haliea sp.* SAOS-164)**	50682	43430	473	MSTVLFRNFRQLVCAGAPGSVLRDVDLCA RDGMITAIGPQLPLTDVDEVVDCGGLTAYP GLVNTHHHFFQALVRNLPGLDWTTLSLLEW LDTIYPIFARLDEDCIYHASLISLADLLKHGCT TAFDHQYNFNSNMGSRVVDRQFEAAALLG ARLHVGRGCNTLPMSAGSTIPDAMLETTDA FLADCERLIGAFHNPAPGAMAQVVVAPCQP VNSLPETFPEAAALARRHGVRLHTHLSEGE NAAMLDRFGMRSLDWCESVGFVGPDVWF AHGWEFTPPEIARLAATGTGVAHCPAPVFL VGAEVTDLPAMVAADMTVGMGVDGQASN DSSNLAECMRLAYLLQCLNARHNPLPAPPP ERYLHMATAGGAACLGRTDIGELAVGKAAD FFCADLNGLDYAGADSDPLSLPAKVGFAGP AAMTVVHGRVVWRDGEFPGLDETQLRSAA DALLREKLDGHLAPLRTPGLEHHHHHH

***po*NdmH (previously[1,3] ‘AtzB_Hom_Pleo’) (from *Pleomorphomonas oryzae*)**	51181	48360	467	MGNYLLKNCAAVMVDDGAGLNARRNVDILT<br>DGPAIKAIEPHLAETPQSVGAEVIDASGWFV<br>YPGLVNTHHHFFQTFVRNRAELDWTKLSVL<br>EWLDRIYPIFSQLTEDCFYHSSLTAMAELIK<br>HGCTTALDHQYCFPRHAGKYLVDRQFEAA<br>ERLGIRYHAGRGGNTLPKSEGSTIPDAMLE<br>TTDEFLADCERLIDRYHDASPFSLRQVVISP<br>CQPVNSYRETFVESVALARDKGVFLHTHVG<br>EGESPVMEARHGKRTVDYLEEMGFAGPDV<br>FYAHCWELTHTELAKLAASGTGVSHCPEPV<br>YLVGAEVTDIPAMAALGVRVGLGCDGSASN<br>DNSNLMHCIHSAYMLQCLVASSRSHPVPAP<br>AEFLRFATTGSASLLGRADIGRLAPGMAAD<br>LFAIDTRRMDYVGTRHDPLSLPAKLGIGMAT<br>DLTMINGRIVWANGEFPGIDEAEMAAEAEA<br>TLATIDFLEHHHHHH
***rb*NdmH (previously[1] ‘MBD1203459’) (from *Rhodobacteraceae bacterium*)**	50303	46870	462	MHHHHHHLDMTAHLFKGCAAVICDPHSVLR<br>DVDLLVEGPKIAAIGKGLVAPTGAEVIDARG<br>WFLYPGLVNTHHHFFQTFVRNRADLDWTK<br>LSVIEWLDLIYPIFSRLTEDCFYHSSLTAMAE<br>LAKHGCTTAFDHQYNYPRHAGKRLVDRQF<br>EAAAKIGLRFHAGRGGNTLPKSQGSTIPDE<br>MLESTDEFIADCARLIDTYHDSAPFSMAQVV<br>VSPCQPVNCYRETFVESAALARDKGVFLHT<br>HVGEGESQVIAARHGMRTVDYLEQIGFAGP<br>DTFYAHCWELTDTELRQLAASGTGVAHCP<br>EPVYLVGAEVTDVPAMAAFGVRLGLGCDG<br>SASSDNSNLMHCIHSGYMLQCLVASRRAH<br>PVPEPRDFLGYATAGGAALLGRSDIGRLAP<br>GMAADLFAIDTRRMDYVGTRHDPASLIAKV<br>GIAMPTDLTMVNGRIVWAKGEFPGLDEAQ<br>MAAEAEAVLATINA

Supporting references

[1] L. Drexler, T. F. Fürtges, T. Rudack, R. Sterner. On the Origin of Substrate Specificity of Enzymes from the Amidohydrolase Superfamily, *Angew. Chem. Int. Ed.* **2026**, *65*, e17873.

[2] N. Oberg, R. Zallot, J. A. Gerlt. EFI-EST, EFI-GNT, and EFI-CGFP: Enzyme Function Initiative (EFI) Web Resource for Genomic Enzymology Tools, *J. Mol. Biol.* **2023**, *435*, 168018.

[3] M. R. Busch, L. Drexler, D. R. Mahato, C. Hiefinger, S. Osuna, R. Sterner. Retracing the Rapid Evolution of an Herbicide-Degrading Enzyme by Protein Engineering, *ACS Catal.* **2023**, *13*, 15558–15571.

[4] R. S. Hall, A. A. Fedorov, R. Marti-Arbona, E. V. Fedorov, P. Kolb, J. M. Sauder, S. K. Burley, B. K. Shoichet, S. C. Almo, F. M. Raushel. The hunt for 8-oxoguanine deaminase, *J. Am. Chem. Soc.* **2010**, *132*, 1762–1763.

[5] J.T. Maynes, R.G. Yuan, F.F. Snyder. Identification, expression, and characterization of Escherichia coli guanine deaminase., *J. Bacteriol.* **2000**, *182*, 4658–4660.

[6] C. M. Seibert, F. M. Raushel. Structural and catalytic diversity within the amidohydrolase superfamily, *Biochemistry* **2005**, *44*, 6383–6391.

[7] E. Sugrue, N. J. Fraser, D. H. Hopkins, P. D. Carr, J. L. Khurana, J. G. Oakeshott, C. Scott, C. J. Jackson. Evolutionary expansion of the amidohydrolase superfamily in bacteria in response to the synthetic compounds molinate and diuron, *Appl. Environ. Microbiol.* **2015**, *81*, 2612–2624.

[8] R. Shek, T. Hilaire, J. Sim, J. B. French. Structural Determinants for Substrate Selectivity in Guanine Deaminase Enzymes of the Amidohydrolase Superfamily, *Biochemistry* **2019**, *58*, 3280–3292.

[9] J. Jumper, R. Evans, A. Pritzel, T. Green, M. Figurnov, O. Ronneberger, K. Tunyasuvunakool, R. Bates, A. Žídek, A. Potapenko, A. Bridgland, C. Meyer, S. A. A. Kohl, A. J. Ballard, A. Cowie, B. Romera-Paredes, S. Nikolov, R. Jain, J. Adler, T. Back, S. Petersen, D. Reiman, E. Clancy, M. Zielinski, M. Steinegger, M. Pacholska, T. Berghammer, S. Bodenstein, D. Silver, O. Vinyals, A. W. Senior, K. Kavukcuoglu, P. Kohli, D. Hassabis. Highly accurate protein structure prediction with AlphaFold, *Nature* **2021**, *596*, 583–589.

[10] J. L. Seffernick, A. Aleem, J. P. Osborne, G. Johnson, M. J. Sadowsky, L. P. Wackett. Hydroxyatrazine N-ethylaminohydrolase (AtzB): an amidohydrolase superfamily enzyme catalyzing deamination and dechlorination., *J. Bacteriol.* **2007**, *189*, 6989–6997.

[11] A. Warshel, P. K. Sharma, M. Kato, Y. Xiang, H. Liu, M. H. M. Olsson. Electrostatic basis for enzyme catalysis, *Chemical reviews* **2006**, *106*, 3210–3235.

[12] J. P. Klinman, S. M. Miller, N. G. J. Richards. A Foundational Shift in Models for Enzyme Function, *J. Am. Chem. Soc.* **2025**, *147*, 14884–14904.

[13] M. Roca, J. Andrés, V. Moliner, I. Tuñón, J. Bertrán. On the nature of the transition state in catechol O-methyltransferase. A complementary study based on molecular dynamics and potential energy surface explorations, *J. Am. Chem. Soc.* **2005**, *127*, 10648–10655.

[14] M. D. Hanwell, D. E. Curtis, D. C. Lonie, T. Vandermeersch, E. Zurek, G. R. Hutchison. Avogadro: an advanced semantic chemical editor, visualization, and analysis platform, *J. Cheminform.* **2012**, *4*, 17.

[15] H. Jabeen, M. Beer, J. Spencer, M. W. van der Kamp, H. A. Bunzel, A. J. Mulholland. Electric Fields Are a Key Determinant of Carbapenemase Activity in Class A β-Lactamases, *ACS Catal.* **2024**, *14*, 7166–7172.

[16] S. D. Fried, S. G. Boxer. Electric Fields and Enzyme Catalysis, *Annual review of biochemistry* **2017**, *86*, 387–415.

[17] C. Zheng, Z. Ji, I. I. Mathews, S. G. Boxer. Enhanced active-site electric field accelerates enzyme catalysis, *Nat. Chem.* **2023**, *15*, 1715–1721.

[18] D. R. Roe, T. E. Cheatham. PTRAJ and CPPTRAJ: Software for Processing and Analysis of Molecular Dynamics Trajectory Data, *J. Chem. Theory Comput.* **2013**, *9*, 3084–3095.

[19] T. Darden, D. York, L. Pedersen. Particle mesh Ewald: An N ·log(N) method for Ewald sums in large systems, *J. Chem. Phys.* **1993**, *98*, 10089–10092.

[20] R. T. McGibbon, K. A. Beauchamp, M. P. Harrigan, C. Klein, J. M. Swails, C. X. Hernández, C. R. Schwantes, L.-P. Wang, T. J. Lane, V. S. Pande. MDTraj: A Modern Open Library for the Analysis of Molecular Dynamics Trajectories, *Biophys. J.* **2015**, *109*, 1528–1532.

[21] M. K. Scherer, B. Trendelkamp-Schroer, F. Paul, G. Pérez-Hernández, M. Hoffmann, N. Plattner, C. Wehmeyer, J.-H. Prinz, F. Noé. PyEMMA 2: A Software Package for Estimation, Validation, and Analysis of Markov Models, *J. Chem. Theory Comput.* **2015**, *11*, 5525–5542.

[22] J. L. Seffernick, A. G. Dodge, M. J. Sadowsky, J. A. Bumpus, L. P. Wackett. Bacterial ammeline metabolism via guanine deaminase., *J. Bacteriol.* **2010**, *192*, 1106–1112.
